# Sex-Specific Features of the Correlation between GWAS-Noticeable Polymorphisms and Hypertension in Europeans of Russia

**DOI:** 10.3390/ijms24097799

**Published:** 2023-04-25

**Authors:** Tatiana Ivanova, Maria Churnosova, Maria Abramova, Denis Plotnikov, Irina Ponomarenko, Evgeny Reshetnikov, Inna Aristova, Inna Sorokina, Mikhail Churnosov

**Affiliations:** 1 Department of Medical Biological Disciplines, Belgorod State National Research University, 308015 Belgorod, Russia; 4602@bsu.edu.ru (T.I.); churnosovamary@gmail.com (M.C.); abramova_myu@bsu.edu.ru (M.A.); ponomarenko_i@bsu.edu.ru (I.P.); reshetnikov@bsu.edu.ru (E.R.); aristova@bsu.edu.ru (I.A.); sorokina@bsu.edu.ru (I.S.); 2 Genetic Epidemiology Lab, Kazan State Medical University, 420012 Kazan, Russia; denis.plotnikov@kazangmu.ru

**Keywords:** sex, blood pressure, hypertension, GWAS, SNP, association

## Abstract

The aim of the study was directed at studying the sex-specific features of the correlation between genome-wide association studies (GWAS)-noticeable polymorphisms and hypertension (HTN). In two groups of European subjects of Russia (*n* = 1405 in total), such as men (*n* = 821 in total: *n* = 564 HTN, *n* = 257 control) and women (*n* = 584 in total: *n* = 375 HTN, *n* = 209 control), the distribution of ten specially selected polymorphisms (they have confirmed associations of GWAS level with blood pressure (BP) parameters and/or HTN in Europeans) has been considered. The list of studied loci was as follows: (*PLCE1*) rs932764 A > G, (*AC026703.1*) rs1173771 G > A, (*CERS5*) rs7302981 G > A, (*HFE*) rs1799945 C > G, (*OBFC1*) rs4387287 C > A, (*BAG6*) rs805303 G > A, (*RGL3*) rs167479 T > G, (*ARHGAP42*) rs633185 C > G, (*TBX2*) rs8068318 T > C, and (*ATP2B1*) rs2681472 A > G. The contribution of individual loci and their inter-locus interactions to the HTN susceptibility with bioinformatic interpretation of associative links was evaluated separately in men’s and women’s cohorts. The men–women differences in involvement in the disease of the BP/HTN-associated GWAS SNPs were detected. Among women, the HTN risk has been associated with *HFE* rs1799945 C > G (genotype GG was risky; OR_GG_ = 11.15 p_permGG_ = 0.014) and inter-locus interactions of all 10 examined SNPs as part of 26 intergenic interactions models. In men, the polymorphism *BAG6* rs805303 G > A (genotype AA was protective; OR_AA_ = 0.30 p_permAA_ = 0.0008) and inter-SNPs interactions of eight loci in only seven models have been founded as HTN-correlated. HTN-linked loci and strongly linked SNPs were characterized by pronounced polyvector functionality in both men and women, but at the same time, signaling pathways of HTN-linked genes/SNPs in women and men were similar and were represented mainly by immune mechanisms. As a result, the present study has demonstrated a more pronounced contribution of BP/HTN-associated GWAS SNPs to the HTN susceptibility (due to weightier intergenic interactions) in European women than in men.

## 1. Introduction

HTN belongs to the group of the most common human diseases, a characteristic feature of which is high BP [1]. According to statistical reports in the world over the past decades (from 1990 to 2015), the number of persons with a systolic BP (SBP) of 140 mm Hg and above rose by 18.59% (from 17,307 to 20,526 per 100 thousand population), and the mortality and disability-adjusted life-years (DALYs) rates associated with an SBP of 140 mm Hg and higher increased by 8.58% (from 97.9 to 106.3 per 100,000 people) and 49.11% (from 95.9 million to 143.0 million), respectively [2]. A rise in SBP by 10 mm Hg leads to an increased risk of coronary heart disease by 45% and ischemic/hemorrhagic stroke by 63–66% in individuals aged 55–64 years [3]. The problem of HTN is no less important for the Russian population [4,5], among which the projected number of deaths and DALYs correlated with SBP ≥ 140 mm Hg increased by 25.6% (from 452.3 to 568.0 thousand) and 49.11% (from 8267.8 to 9672.7 thousand) over the period from 1990 to 2015, respectively [2]. The disease is more often (≈20%) registered in men than in women (age-standardized prevalence rates of HTN in men and women are 24 and 20%, respectively) [6] and this pattern is also characteristic of the population of Russia [5]. In men, the indicator DALYs linked with SBP ≥ 140 mmHg (82.915 million) significantly exceeds (1.38 times) the same parameter in women (60.122 million) [2]. Even more pronounced differences (by 1.44 times) between men (125.124 million) and women (86.692 million) are observed in the value for DALYs correlated with SBP ≥ 110–115 mm Hg [2]. Significant differences in the prevalence of HTN and its impact on the state of health in men and women may be associated with both hormonal mechanisms [7] and environmental risk factors [8], which determine the features of gene–environmental interactions [9,10].

The influence of hereditary factors on the HTN occurrence has been studied for a long time and, at present, the genetic contribution to the disease formation has been confirmed in numerous works performed on the basis of twin/family [11,12,13,14,15,16,17], GWAS ([9,10,18,19,20,21,22,23,24,25,26,27,28,29,30,31,32,33,34], etc.) and other associative (based on the candidate genes’ analysis) ([35,36,37,38,39,40,41,42,43,44,45,46,47,48], etc.) studies. The prevailing value of genetic factors in HTN development is confirmed by the fact that the presence of a family history burden increases the disease risk by 3.5–3.8 times compared to the general population [49,50]. Estimates of the heredity contribution to BP vary on average from 30% to 55% [11,12,14,15,16,17], reaching the level of 63–68% in some works [12,13]. At the same time, the BP heritability indicators, estimated on the basis of currently known GWAS data (SNP-wide heritability), amount to 19.4–21.3% [51] and only partially “reveal” genetic factors of HTN predisposition (from 1/3 to 2/3). In general, the currently known loci, detected by GWAS, explain only 2.87–5.66% of BP variance [51]. The above data indicate the presence of a problem of “hidden” (unidentified) heredity for BP (and, accordingly, HTN) [16] and dictate the need for further research aimed at solving it.

So, the available literature materials clearly indicate, on the one hand, a significant heredity contribution to HTN susceptibility [11,12,14,15,16,17]; but, on the other hand, demonstrate pronounced men–women differences in HTN incidence [4,6]. These facts stipulate the appropriateness of genetic and epidemiological studies “revealing” the genetic mechanisms underlying the differences in the genetic determination of HTN in men and women (as one of the factors determining sex-specific differences in the disorder incidence), including using genes/polymorphisms with a previously proven strong role (GWAS data) in HTN predisposition. In some previously performed studies (including the European population of Russia investigated in this paper), men–women differences in the involvement in the formation of HTN polymorphisms of a number of candidate genes (matrix metalloproteinases, tumor necrosis factors, etc.) are shown, including the taking into account of the action of sex-specific risk factors of the disease such as obesity, smoking, etc. [40,45,46,47,48] (etc.). In addition, significant men–women differences in sex hormone genetic traits [52,53,54], BMI genetic traits (obesity, waist/hip circumference, etc.) [55,56,57,58,59] and insulin/type 2 diabetes genetic traits [59,60] have been demonstrated at the GWAS level, which may be remarkable genetic predictors of sex-specific differences for cardiovascular diseases [61,62,63,64,65].

Our study was directed at studying the sex-specific features of the correlation between GWAS-noticeable polymorphisms and HTN.

## 2. Results

The characteristics of generalized participants (men and women) have been given in the Table 1. In both men (*n* = 821) and women (*n* = 584) cohorts, HTN subjects differed from the HTN-free subjects by higher values of body mass index (BMI), total cholesterol (TC), triglycerides (TG), low-density lipoprotein cholesterol (LDL-C), blood glucose, smokers and a lower high-density lipoprotein cholesterol (HDL-C) level (*p* < 0.05–0.001). Based on these results, the aforementioned features were introduced into genetic calculations as covariates in both men and women (Model 1). There were also differences between patients and controls (both in men and women) in such parameters as low physical activity and high fatty food consumption (in patients, these indicators were significantly higher, *p* < 0.001). In order to assess the effect of these indicators of lifestyle (physical activity) and diet (fatty food consumption) on genetic associations, we additionally included them in the analysis as confounders in Model 2.

The SNPs allele and genotype frequencies in the HTN and HTN-free groups of men (Table S1) and women (Table S2) have Hardy–Weinberg (H-W) equilibrium state, p_Bonferroni_ ≥ 0.025 (Bonferroni’s correction related to the amount of comparison pairs studied was accounted for, *n* = 2 [men and women]).

The men–women differences in involvement in the disease of the BP/HTN-associated GWAS SNPs were detected (both in Model 1 and Model 2). According to Model 1, among men, the polymorphism *BAG6* rs805303 G > A was founded as HTN-correlated (genotype AA was protective; OR_AA_ = 0.30; p_AA_ = 0.0008; p_permAA_ = 0.0008 [recessive model] statistical power = 98.75%) (Table 2). In women, the HTN risk was associated with *HFE* rs1799945 C > G (genotype GG was risky; OR_GG_ = 11.15; p_GG_ = 0.011; p_permGG_ = 0.014 [recessive model] statistical power = 99.99%) (Table 2). Almost similar results were obtained in Model 2: the SNP *BAG6* rs805303 G > A was HTN-associated in men (OR_AA_ = 0.31; p_AA_ = 0.001; p_permAA_ = 0.001; statistical power = 98.43% [recessive model]) (Table 2) and locus *HFE* rs1799945 C > G was HTN-involved in women (OR_GG_ = 10.96; p_GG_ = 0.012; p_permGG_ = 0.014; statistical power = 99.99% [recessive model]) (Table 2). The great similarity in the results of our evaluation of genetic associations in Model 1 and Model 2 may be due to the fact that, apparently, the effects of additional confounders included in Model 2 (low physical activity and high fatty food consumption) have already been “taken into account” in the HTN effects of confounders of Model 1. For example, the high consumption of fatty foods (confounder Model 2) obviously directly determines the lipid profile of the body such as TC, TG, LDL-C and HDL-C levels (confounders Model 1), and low physical activity (confounder Model 2) will largely correlate with BMI (confounder Model 1). Accordingly, at the next stage of the work, when analyzing the associations of inter-locus interactions with HTN, we used a list of confounders of Model 1.

Pronounced sex-specific differences in engagement in the HTN of GWAS-noticeable polymorphisms were revealed during the evaluation of inter-locus communications. In the men’s group the inter-SNP interactions of eight loci in only seven models have been registered as HTN-involved (p_perm_ ≤ 0.022, polymorphic loci of two genes—*ARHGAP42* rs633185 C > G, *ATP2B1* rs2681472 A > G, not included in these models) (Table 3). Polymorphisms of four genes such as *CERS5* rs7302981 G > A, *AC026703.1* rs1173771 G > A, *HFE* rs1799945 C > G and *PLCE1* rs932764 A > G were represented in more than 50% of all models. The best possible genetic model is a four-gene model (*PLCE1* rs932764 A > G × *CERS5* rs7302981 G > A × *HFE* rs1799945 C > G × *TBX2* rs8068318 T > C) with Wald stat. = 32.12 (p_perm_ = 0.001). Twenty-three HTN-associated combinations of genotypes were established in men, practically all of which were disorder-protective (22 out of 23, 95.65%). Only one genotype combination (three SNPs) such as rs805303-GG (*BAG6*) × rs1173771-AA (*AC026703.1*) × rs4387287-AA (*OBFC1*) increases the HTN risk in men (beta = 2.29; *p* = 0.029). Among the HTN-protective combinations of genotypes, the brightly expressed effect (*beta* values were maximum) has been demonstrated by rs932764-GG (*PLCE1*) × rs8068318-TC (*TBX2*) × rs805303-GG (*BAG6*) × rs1173771-GA (*AC026703.1*) (*beta* = −2.63; *p* = 0.017), rs7302981-GG (*CERS5*) × rs1799945-CC (*HFE*) × rs805303-GG (*BAG6*) × rs167479-GG (*RGL3*) (*beta* = −2.24; *p* = 0.042), rs932764-GG (*PLCE1*) × rs7302981-GA (*CERS5*) × rs1799945-CG (*HFE*) × rs8068318-CC (*TBX2*) (*beta* = −2.13; *p* = 0.002), rs932764-AA (*PLCE1*) × rs7302981-GG (*CERS5*) × rs1799945-CC (*HFE*) × rs1173771-GA (*AC026703.1*) (*beta* = −2.01; *p* = 0.019) (Table S3).

In the women’s cohort, the HTN genetic risk was associated with inter-locus interactions of all 10 examined SNPs as part of 26 intergenic interaction models (p_perm_ ≤ 0.016) (Table 3). Three genes/polymorphisms such as *RGL3* rs167479 T > G (it is included in 18 models), *TBX2* rs8068318 T > C (16 models), *HFE* rs1799945 C > G (13 models) make the prevailing contribution to HTN susceptibility (they were part of 50% of models and more). The four-gene/locus model, including *PLCE1* rs932764 A > G × *TBX2* rs8068318 T > C × *AC026703.1* rs1173771 G > A × *RGL3* rs167479 T > G, is the top (Wald stat. = 33.53; p_perm_ < 0.001). Ninety-four different HTN-associated genotypes combinations were modeled (Table S4). Among them, the large majority of genetic combinations (*n* = 67, 71.28%) have a protective orientation (*beta* < 0) and only less than 1/3 combinations (*n* = 27, 28.72%) are risky (*beta* > 0). Genotype combinations with the maximum phenotypic effects in relation to the HTN risk (characterized by the highest *beta* indicators) were established both up-predisposition (risky) [rs932764-AA (*PLCE1*) × rs8068318-TC (*TBX2*) × rs167479-GG (*RGL3*) (*beta* = 2.45; *p* = 0.024)] and down-predisposition (protective) [rs633185-GG (*ARHGAP42*) × rs932764-GG (*PLCE1*) × rs7302981-GA (*CERS5*) × rs805303-GA (*BAG6*) (*beta* = −3.11; *p* = 0.003); rs932764-AG (*PLCE1*) × rs1799945-GG (*HFE*) × rs8068318-CC (*TBX2*) × rs167479-TG (*RGL3*) (*beta* = −2.88; *p* = 0.012); rs932764-AG (*PLCE1*) × rs1799945-CG (*HFE*) × rs8068318-CC (*TBX2*) (*beta* = −2.87; *p* = 0.012), rs932764-AG (*PLCE1*) × rs1799945-GG (*HFE*) × rs1173771-GA (*AC026703.1*) × rs167479-TG (*RGL3*) (*beta* = −2.79; *p* = 0.016)] orientation.

**Figure 1:**
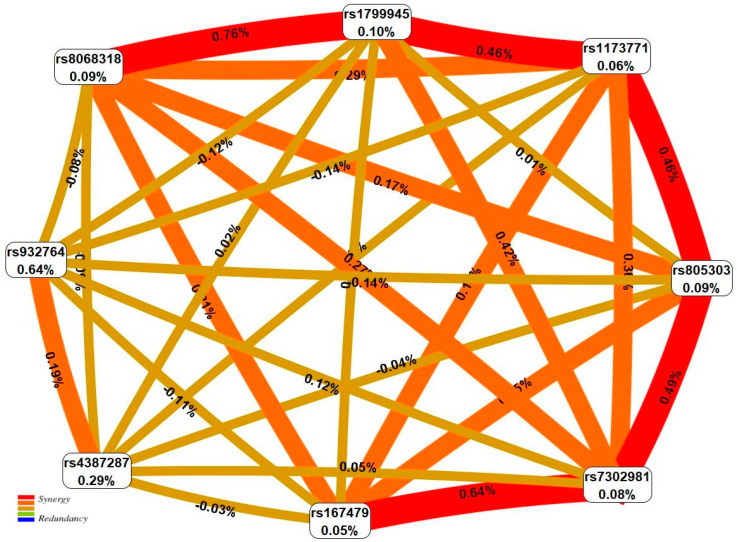
The entropy graph of the SNP×SNP interactions with HTN in men based on the MDR analysis. Positive values of entropy indicate synergistic interactions while the negative values indicate redundancy. The red and orange colors denote strong and moderate synergism, respectively, the brown color denotes the independent effect, the green and blue colors denote moderate and strong antagonism.

**Figure 2:**
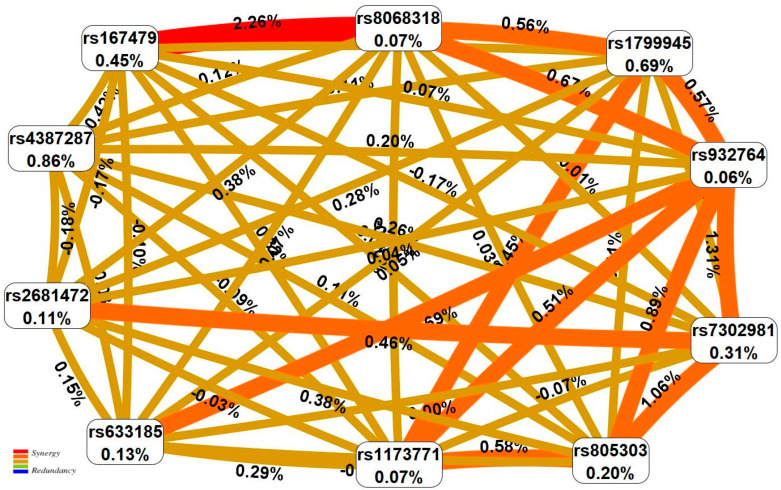
The entropy graph of the SNP×SNP interactions with HTN in women based on the MDR analysis. Positive values of entropy indicate synergistic interactions while the negative values indicate redundancy. The red and orange colors denote strong and moderate synergism, respectively, the brown color denotes the independent effect, the green and blue colors denote moderate and strong antagonism.

The results of the MDR analysis showed that the greatest contribution to the HTN susceptibility in men was made by two-gene/locus synergetic interactions *HFE* (rs1799945)–*TBX2* (rs8068318) (0.76% of HTN entropy), *CERS5* (rs7302981)–*RGL3* (rs167479) (0.64%), *CERS5* (rs7302981)–*BAG6* (rs805303) (0.49%), *BAG6* (rs805303)–*AC026703.1* (rs1173771) (0.46%), *HFE* (rs1799945)–*AC026703.1* (rs1173771) (0.46%) (Figure 1). In women, the HTN predisposition was determined by the strongly pronounced influence of three two-gene/locus synergetic interactions *TBX2* (rs8068318)–*RGL3* (rs167479) (2.26%), *PLCE1* (rs932764)–*CERS5* (rs7302981) (1.31%) and *CERS5* (rs7302981)–*BAG6* (rs805303) (1.06%) (Figure 2). Interestingly, the percentage of disorder entropy explained by the HTN-important pair interactions between genes/loci in women (1.06–2.26%) is significantly higher (2–3 times) than in men (0.46–0.76%), which indicates a more significant role of intergenic/inter-locus interactions in the HTN formation in women compared to men.

### 2.1. Intended Functionality of HTN-Associated SNPs in Men and Women Cohorts

In this section of our work, we evaluated the alleged functionality of all 10 GWAS loci examined and 125 strongly linked SNPs associated with HTN in women (in total, information about 135 loci was considered), and 8 GWAS loci and 96 LD SNPs correlated with HTN in men (data on 104 loci were studied).

#### 2.1.1. Prediction of the Possible SNPs Link with Amino Acid Substitution and Resulting with Human Protein Structure/Function

Among the HTN-involved loci in both men and women, nucleotide changes [T > G (rs167479); C > G (rs1799945); G > A (rs7302981); G > A (rs1046089); and C > G (rs1057987)] in the exons of the five genes (RGL3; HFE; CERS5; PRRC2A; TBX2, respectively) were non-synonymous and led to certain substitutions of amino acids (P162H; H63D; C9R; R1740H; S609R, respectively) in five corresponding proteins with potentially different predictor capability such as “deleterious/probably damaging” (SIFT/PolyPhen categories) [P162H; R1740H/P162H; H63D; R1740H] and “tolerated/benign” (SIFT/PolyPhen categories) [H63D; C9R; S609R/C9R; S609R] (Table S5).

#### 2.1.2. Epigenetic Changes of DNA Determined by HTN-Related Loci

Haploreg epigenomic annotations were used to identify regulatory variants among HTN-related loci in men (*n* = 104) and women (*n* = 135). The proposed functionality of the enormous proportion of analyzed loci [100/104, 96.15% (men); 130/135, 96.29% (women)] was found (Table S6), and these loci were located in such functionally active DNA sequences as enhancers [51/104, 49.04% (men); 69/135, 51.11% (women)], promoters [30/104, 28.85% (men); 30/135, 22.22% (women)], DNAase hyper-sensitive chromatin [47/104, 45.19% (men); 64/135, 47.41% (women)], evolutionary conservation nucleotide sequences [14/104, 13.46% (men); 19/135, 14.07% (women)], sites of DNA binding to regulatory proteins [23/104, 22.12% (men); 29/135, 21.48% (women)] and transcription factors [87/104, 83.65% (men); 113/135, 83.70% (women)]. More than half of all estimated loci were located in introns [62/104, 59.62% (men); 75/135, 55.55% (women)], about 5% in coding regions of the genome [6/104, 5.77% (men); 6/135, 4.44% (women)], about 4% in 3′-UTR gene regions [5/104, 4.81% (men); 5/135, 3.70% (women)] and less than 1% in 5′-UTR gene regions of the genome [1/104, 0.96% (men); 1/135, 0.74% (women)] (Table S6).

In aggregate, HTN-significant loci were functional with respect to 25 genes in men (AC026703.1; BAG6; CERS5; BCAS3; C12orf62; COX14; GPD1; CSNK2B; HIST1H2AC; GPD1; HFE; HIST1H1T; HIST1H2BC; LIMA1; HIST1H4C; PRRC2A; LY6G5B; RP11-411N4.1 LASS5; NPR3; PLCE1; RGL3; OBFC1; TBX2; C17orf82) and 30 genes in women (BCAS3; HIST1H1T; ATP2B1; AC026703.1; BAG6; ARHGAP42; CERS5; C12orf62; CSNK2B; COX14; GPD1; NPR3; HIST1H2AC; GPD1; LASS5; HFE; HIST1H2BC; LIMA1; HIST1H4C; LOC338758; PLCE1; RP11-981P6.1; LY6G5B; RGL3; OBFC1; POC1B-GALNT4; PRRC2A; RP11-411N4.1; TBX2; C17orf82) (Table S6).

The connection of several HTN-causal genes/loci such as *AC026703.1* rs1173771 G > A, *CERS5* rs7302981 G > A, *OBFC1* rs4387287 C > A, *BAG6* rs805303 G > A with the DNA epigenetic modifications characteristic of promoters (H3K4me3/H3K9ac) and enhancers (H3K4me1/H3K27ac) in disorder target organs—heart (fetal [rs4387287; rs7302981] and adult [rs1173771; rs805303; rs4387287; rs7302981]), aorta [rs4387287], is very interesting and important for understanding the involvement of the investigated polymorphisms in the HTN pathophysiology.

#### 2.1.3. Expression Quantitative Traits (eQTL) Associated with HTN-Significant SNPs

The materials of the Blood eQTL browser specify the connection of a number of HTN-significant loci (four causal SNPs [*CERS5* rs7302981 G > A, *HFE* rs1799945 C > G, *OBFC1* rs4387287 C > A, *BAG6* rs805303 G > A] and 23 LD SNPs in men and five causal SNPs [*CERS5* rs7302981 G > A, *HFE* rs1799945 C > G, *OBFC1* rs4387287 C > A, *BAG6* rs805303 G > A, *ATP2B1* rs2681472 A > G] and 33 proxies in women) with the mRNA production of several genes (nine genes in men [*LY6G5C*, *SLK*, *LIMA1*, *HCP5*, *HSPA1B*, *LASS5*, *AIF1*, *TRIM38*, *ALAS2,*] and ten genes in women [*WDR51B*, *LIMA1*, *LASS5*, *LY6G5C*, *SLK*, *AIF1*, *TRIM38*, *ALAS2*, *HCP5*, *HSPA1B*]) in blood (Tables S7 and S8).

The data from a comprehensive public GTEx resource show a tissue-specific link between HTN-involved SNPs and gene expression in non-diseased tissue. All 8 HTN-causal SNPs and 88 of 96 coupled loci (91.67%) in men, and all 10 HTN-causal SNPs and 114 out 125 linked loci (91.20%) in women have allele-specific eQTL effects (Tables S9 and S10). The HTN-impact eQTL-dependent gene list in men and women has considerable similarities. Eighty HTN-associated genes due to the eQTL effects of HTN-related loci are the same in men and women. These are genes such as *NPR3*, *NEU1*, *NCR3*, *MPIG6B*, *MIR6891*, *MICB*, *LY6G6F*, *LY6G6E*, *LY6G6D*, *CYP21A2*, *CYP21A1P*, *CTC-510F12.3*, *CSNK2B*, *LY6G5C*, *LY6G5B*, *LIMA1*, *LARP4*, *ZNF322*, *ZBTB12*, *XXbac-BPG248L24.12*, *WASF5P*, *VWA7*, *UQCRHP1*, *U91328.19*, *TRIM38*, *TBX2-AS1*, *TBX2*, *STN1*, *STK19B*, *STK19*, *SMARCD1*, *SLK*, *SLC17A3*, *RNF5*, *SLC17A1*, *SH3PXD2A-AS1*, *SH3PXD2A*, *RP4-605O3.4*, *RP3-405J10.3*, *RP11-541N10.3*, *RP11-457M11.5*, *RGL3*, *PRRC2A*, *POU5F1*, *HSPA1A*, *HLA-S*, *HLA-DRB6*, *HLA-DRB5*, *HLA-DQB1*, *HLA-B*, *HIST1H3E*, *HFE*, *HDAC1P1*, *HCP5*, *HCG22*, *GUSBP2*, *GSTO1*, *GPD1*, *GPANK1*, *DXO*, *DIP2B*, *DDAH2*, *COX14*, *CLIC1*, *CERS5*, *ATF6B*, *ATF1*, *ASIC1*, *AQP5*, *APOM*, *AIF1*, *BAG6*, *C4B*, *C4A*, *CCHCR1*, *C6orf48*, *BTN2A3P*, *ATP6V1G2*, *LY6G6C*, *ABHD16A*. Nine genes such as *TMEM133*, *RP11-981P6.1*, *ARHGAP42*, *POC1B-AS1*, *ATP2B1*, *GALNT4*, *ATP2B1-AS1*, *POC1B*, *RP11-567C2.1* are added to the HTN-impact genes list in women due to the eQTL effects of two additional HTN-related polymorphisms (*ARHGAP42* rs633185 C > G, *ATP2B1* rs2681472 A > G).

We found interesting SNP-eQTL correlations in organs (heart/aorta/coronary arteries, etc.) that are targets for HTN. For example, among HTN-causal SNPs with gene transcription in the heart have been associated rs1799945 (*HIST1H3E*), rs805303 (*LY6G5B*, *HLA-DRB5*, *DDAH2*, *CSNK2B*, *C4A*, *CYP21A1P*), rs633185 (*ARHGAP42*, *TMEM133*), rs7302981 (*RP4-605O3.4*, *CERS5*), rs2681472 (*RP11-981P6.1*), rs8068318 (*TBX2-AS1*, *TBX2*) (Table S9). In another example, SNP-eQTL connections in the aorta demonstrated loci such as rs1799945 (*HFE*), rs805303 (*LY6G5B*, *HLA-DRB5*, *BAG6*, *LY6G5C*, *ABHD16A*, *VWA7*, *C4A*, *CYP21A1P*), rs633185 (*ARHGAP42*, *TMEM133*), rs7302981 (*ATF1*, *RP4-605O3.4*, *COX14*, *SMARCD1*), rs2681472 (*RP11-981P6.1*, *ATP2B1*), rs8068318 (*TBX2-AS1*) (Table S9). Another clear example of SNP-eQTL correlations significant for the HTN pathophysiology is the connection of some disease-causal loci with mRNA levels in coronary arteries: rs805303 (*LY6G5B*, *LY6G5C*, *BAG6*, *HLA-DRB5*), rs633185 (*ARHGAP42*, *TMEM133*), rs7302981 (*RP4-605O3.4*) (Table S9).

Strongly coupled loci also have significant eQTL potential in the above-mentioned HTN target organs (Table S10). Linked SNPs have a special eQTL significance in relation to 18 genes in the heart (*TMEM133; TBX2-AS1; TBX2; STN1; STK19B; RP4-605O3.4; RP11-981P6.1; NCR3; LY6G5B; HLA-DRB5; HIST1H3E; DDAH2; CYP21A1P; CERS5; CSNK2B; C4A; ATF6B; ARHGAP42*), 21 genes in the aorta (*VWA7; TMEM133; TBX2; TBX2-AS1; SMARCD1; SLK; RP4-605O3.4; RP11-981P6.1; LY6G5C; LY6G5B; HLA-DRB6; HLA-DRB5; HFE; CYP21A1P; COX14; C4A; BAG6; ATP2B1; ATF1; ARHGAP42; ABHD16A*), 7 genes in coronary arteries (*TMEM133; RP4-605O3.4; LY6G5B; HLA-DRB5; BAG6; ARHGAP42; LY6G5C*), 24 genes in other arteries (*ZNF322; TMEM133; TBX2-AS1; TBX2; STK19; SLK; RP4-605O3.4; RP11-981P6.1; POC1B; LY6G5C; LY6G5B; LIMA1; HLA-DRB5; HFE; CYP21A2; C4A; BAG6; COX14; CYP21A1P; ATP2B1-AS1; ATP2B1; ATF1; ARHGAP42; ABHD16A*) (Table S10).

#### 2.1.4. Splicing Quantitative Traits (sQTL) Associated with HTN-Significant SNPs

The presumable splicing regulation by investigated heritable DNA variations were detected. The tissue-specific SNP-splicing associations were recognized for five HTN-causal loci [*CERS5* rs7302981 G > A, *HFE* rs1799945 C > G, *OBFC1* rs4387287 C > A, *BAG6* rs805303 G > A, *TBX2* rs8068318 T > C] and 65 of 96 proxies SNPs (67.71%) in men and six HTN-causal polymorphisms [*CERS5* rs7302981 G > A, *HFE* rs1799945 C > G, *OBFC1* rs4387287 C > A, *BAG6* rs805303 G > A, *TBX2* rs8068318 T > C, *ATP2B1* rs2681472 A > G] and 67 out 125 linked loci (53.60%) in women (Tables S11 and S12). The HTN-significant sQTL-dependent gene list in men (78 genes) and women (79 genes) has an impressive likeness due to the similarity of the sQTL-correlated polymorphism list (five of six loci were the same). A list of the same sQTL-dependent genes for men and women such as (*n* = 78) (*VARS; TBX2-AS1; TBX2; STK19B; STK19; SMARCD1; SH3PXD2A-AS1; RP4-605O3.4; RP11-332H18.9; RP11-332H18.8; RP11-332H18.7; RP11-332H18.6; RP11-332H18.5; RP11-332H18.47; GPANK1; RP11-332H18.46; RP11-332H18.45; RP11-332H18.44; RP11-332H18.43; RP11-332H18.42; RP11-332H18.41; RP11-332H18.40; RP11-332H18.39; RP11-332H18.38; RP11-332H18.37; RP11-332H18.36; RP11-332H18.35; RP11-332H18.34; RP11-332H18.33; RP11-332H18.32; RP11-332H18.31; RP11-332H18.30; RP11-332H18.29; RP11-332H18.28; RP11-332H18.27; RP11-332H18.26; RP11-332H18.25; RP11-332H18.24; RP11-332H18.23; RP11-332H18.22; RP11-332H18.21; RP11-332H18.20; RP11-332H18.19; RP11-332H18.18; RP11-332H18.17; RP11-332H18.16; RP11-332H18.15; RP11-332H18.14; RP11-332H18.13; RP11-332H18.12; RP11-332H18.11; RP11-332H18.10; PRRC2A; MICA; LY6G6C; LY6G5C; LY6G5B; LST1; LSM2; HLA-DRB6; HLA-DRB5; HLA-DRB1; HLA-DQA1; HFE; FLOT1; FAM186A; DDX39B; COX14; CYP21A2; CYP21A1P; CERS5; CCHCR1; C6orf48; BCAS3; BAG6; ATF6B; ATF1; AIF1*) (Tables S11 and S12). In women, only one additional sQTL dependent gene (*POC1B-AS1*) was added to this list.

Multiple SNP-sQTL connections, which are quite essential for HTN pathophysiology, were identified by us as they manifest themselves in organs that are targeted for HTN, such as the heart (rs805303 [*GPANK1; HLA-DRB1; STK19B; HLA-DRB5; LY6G5C; HLA-DRB6; LY6G5C*], rs7302981 [*CERS5*], rs8068318 [*RP11-332H18.13; RP11-332H18.14; RP11-332H18.19; RP11-332H18.20; RP11-332H18.44*]), aorta (rs805303 [*LSM2; BAG6; HLA-DRB1; STK19B; HLA-DRB5; HLA-DRB6*], rs7302981 [*CERS5; COX14; RP4-605O3.4*], rs8068318 [*RP11-332H18.5; RP11-332H18.6*]), coronary artery (rs805303 [*BAG6; HLA-DRB1; HLA-DRB5; HLA-DRB6*], rs7302981 [*CERS5; COX14; RP4-605O3.4*], rs8068318 [*RP11-332H18.11; RP11-332H18.12*]), other arterial vessels (rs805303 [*ATF6B; BAG6*, *GPANK1; HLA-DRB1; HLA-DRB5; HLA-DRB6*], rs7302981 [*CERS5*]), rs8068318 [*RP11-332H18.5; TBX2-AS1*]) (Table S11). More than 60 linked loci also exhibit their sQTL effects in target organs: the heart (ten genes such as *TBX2-AS1; TBX2; STK19B; RP11-332H18.5; LY6G5C; HLA-DRB6; HLA-DRB5; HLA-DRB1; GPANK1; CERS5*), aorta (twelve genes such as *TBX2-AS1; TBX2; STK19B; RP4-605O3.4; RP11-332H18.5; LSM2; HLA-DRB6; HLA-DRB5; BAG6; HLA-DRB1; COX14; CERS5;*), coronary artery (nine genes such as *TBX2-AS1; RP4-605O3.4; RP11-332H18.5; HLA-DRB6; HLA-DRB5; HLA-DRB1; COX14; CERS5; BAG6*), and other arterial vessels (thirteen genes such as *TBX2-AS1; TBX2; RP4-605O3.4; RP11-332H18.5; HLA-DRB6; HLA-DRB5; HLA-DRB1; GPANK1; COX14; CERS5; CCHCR1; BAG6; ATF6B*) (Table S12).

#### 2.1.5. HTN-Associated Gene Pathways

As a result of the evaluation of the functionality at HTN-correlated loci (135 SNPs in women [10 causal loci and 125 strongly linked SNPs] and 104 SNPs in men [8 causal loci and 96 LD SNPs]), 159 genes were found to be involved in the HTN susceptibility in women and 147 genes in men. The substantial similarity in the HTN-involved genes list in men and women determines the almost identical biological pathways in them [in total, using Gene Ontology enrichment analysis tools, more than 140 different pathways were identified in both men (*n* = 145, Table S13) and women (*n* = 141, Table S14)] and were largely represented by pathways associated with the involvement of many different immune reactions/processes. Among both men and women, two biological pathways had the highest rates of statistical significance: MHC (major histocompatibility complex) protein (PANTHER Protein Class ID 00149) (p_(fdr)_ equal 5.30 × 10^−11^ in men and 8.33 × 10^−11^ in women) and antigen processing & presentation (PANTHER Slim Biological Process ID 0019882) (p_(fdr)_ equal 6.00 × 10^−10^ in men and 9.44 × 10^−10^ in women) (Tables S13 and S14).

**Figure 3:**
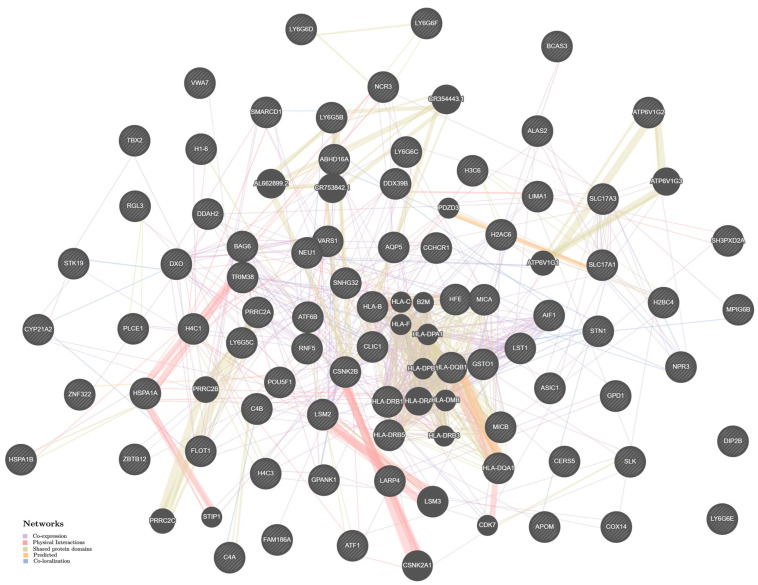
The interaction networks of the candidate genes for HTN in men in various tissues/organs inferred using GeneMANIA (http://genemania.org (accessed on 19 December 2022)). The candidate genes are cross-shaded.

**Figure 4:**
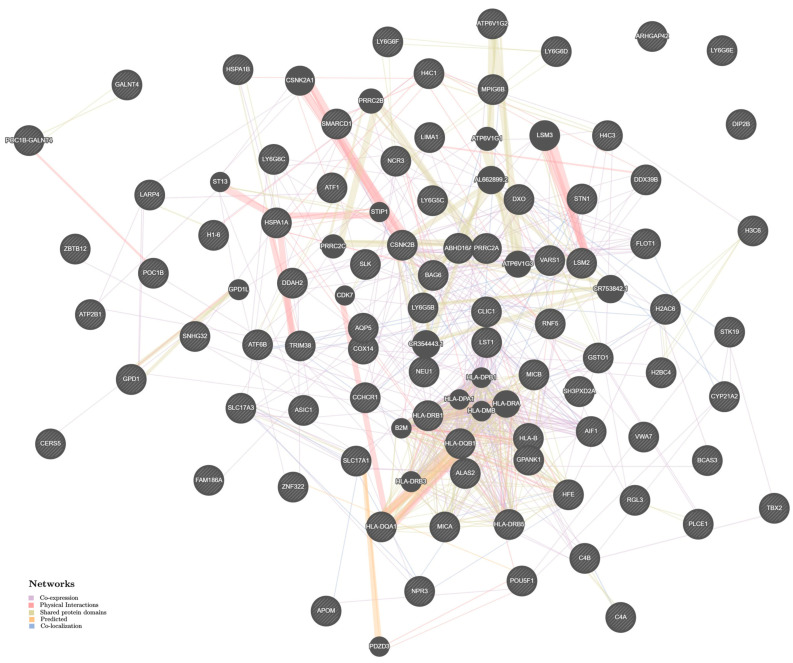
The interaction networks of the candidate genes for HTN in women in various tissues/organs inferred using GeneMANIA (http://genemania.org (accessed on 19 December 2022)). The candidate genes are cross-shaded.

The estimates of the mechanisms of intergenic interactions of HTN-significant genes in men and women, derived with help from the Genemania bioinformatic resource, also turned out to be almost the same. In both men (Figure 3) and women (Figure 4), the main biological mechanisms of the influence of HTN-significant genes on the disease development were co-expression [49.29% (men); 48.28% (women)], physical interactions [29.93% (men); 31.09% (women)], common protein domains [7.68% (men); 7.69% (women)], co-localization [7.33% (men); 7.10% (women)], predicted interactions [5.77% (men); 5.84% (women)]. Cooperation between genes such as *HLA-DQB1—HLA-DQA1*, *CSNK2A1—CSNK2B*, *LSM3—LSM2*, demonstrated the very strong contribution (their weight coefficient was maximum and equal to 1) to the HTN susceptibility in both men (Table S15) and women (Table S16).

## 3. Discussion

The present study identified the men–women differences in Europe in the HTN involvement of the BP/HTN-associated GWAS SNPs. Among women, the HTN risk was determined by *HFE* rs1799945 C > G and inter-locus interactions of all 10 examined SNPs as part of 26 intergenic interactions models, whereas in men, the locus *BAG6* rs805303 G > A and inter-SNPs interactions of eight loci in only seven models were correlated with HTN. The strongly pronounced functionality of HTN-correlated loci (135 SNPs in women [10 causal loci and 125 strongly related SNPs] and 104 SNPs in men [8 causal loci and 96 LD SNPs]) determines the involvement of 159 genes in women and 147 genes in men in the disease susceptibility architecture. A significant similarity in the list of genes involved in HTN in men and women determines almost identical signaling pathways (mainly due to immune mechanisms) in them.

The results of our study showed that the presence of the GG genotype (rs1799945 *HFE*) in a woman substantially increases the chance of HTN developing by more than 10 times (OR-11.15). The HTN-dangerous (high BP) effect of allele G and HTN-safe value (low BP) of allele C polymorphism *HFE* rs1799945 C > G have been detected in earlier studies (GWAS and etc.) [9,18,66,67,68,69,70], which is undividedly compatible with our data in the women’s cohorts.

The polymorphism HFE rs1799945 C > G and the seven proxy loci exhibit pronounced functionality in relation to fifteen genes (U91328.19; RP11-457M11.5; HIST1H4C; ALAS2; SLC17A3; HIST1H1T; BTN2A3P; SLC17A1; HIST1H2AC; GUSBP2; ZNF322; HFE; HIST1H3E; TRIM38; HIST1H2BC) (our in silico data) and are of paramount importance in the regulation of iron metabolism (serum concentration of such iron status biomarkers as iron/transferrin/ferritin/transferrin saturation, total iron binding capacity) (literary GWAS data [71,72]) and related metabolic pathways which are HTN-important (hemoglobin concentration, red cells parameters, glucose homeostasis, glycated hemoglobin levels, etc.) [72,73,74,75,76,77,78,79]. SNP HFE rs1799945 C > G has been correlated with medication agents acting on the renin–angiotensin system [80]. Importantly, Gill et al. used a Mendelian randomization analysis of GWAS (48, 972 European/Genetics of Iron Status Consortium) and PheWAS (424, 439 European/UK Biobank) summary data and founded a causal link between genetically determined levels of serum iron and a hazard of hypercholesterolemia and anemia [81]. The risk value of hypercholesterolemia for HTN (and in common for cardiovascular diseases) is well-known [6,82] and it is also revealed in the sample studied in this work. So, data from the literature and this study material displayed visible HTN-impact pleiotropic effects of HFE rs1799945 C > G.

In the studied men, the genotype AA of *BAG6* rs805303 G > A dramatically reduced the danger of HTN (OR = 0.30). In two early GWAS allele G rs805303 was HTN/high BP-risky and allele A was linked with low BP [18,76]. The higher systolic/diastolic BP in Ugandan adolescents having allele G rs805303 was detected by Lule et al. [83]. So, it can be noted that the same orientation of allelic variants *BAG6* rs805303 G > A is associated with HTN/BP in our study and previously performed works. According to the in silico study results, the locus (with ten proxy SNPs) is the major regulator (the so—called “master regulator”) of epigenetic/expression/splicing traits at fifty-five genes, including immune system genes (e.g., *HLA*, *LY6*, *HSP* gene clusters) which strongly correlated with HTN [84,85,86,87,88] (detailed information about the connection of the immunity-importance above-mentioned genes with HTN is given below).

Large-scale epidemiological studies indicate the presence of visible men–women differences in BP [2,6,8,82,89,90]. At that, these differences were more noticeable in high-income countries and those in Central/Eastern Europe than in the countries of other regions [89]. It is believed that the incidence of HTN in men is 1.2 times higher in comparison with women and the DALYs linked with SBP ≥ 140 mmHg (1.38 times) and SBP ≥ 110–115 mmHg (1.44 times) are greater by1.4 times [2,6]. One of the reasons determining the higher incidence of HTN in men (in comparison with women) may be the prevalence of such risk factors for cardiovascular diseases (including HTN) in them as a diet high in sodium and low in fruit, smoking, alcohol and drug use, and high fasting blood glucose levels [8]. Thus, a more pronounced exposure of environmental risk factors in men than in women, on the one hand, may have an independent HTN risk value, while on the other hand have a significant modifying effect on the realization of hereditary predisposition to the disease. The significant role of gene–environment interactions (SNPs with smoking, alcohol intake) in the nature of candidate gene polymorphism associations with HTN, including those considered in this work *ARHGAP42* rs633185 C > G, *HFE* rs1799945 C > G, *AC026703.1* rs1173771 G > A was shown in previously conducted GWAS [9,10,65]. It should be noted that a number of the above-mentioned HTN risk factors involved in SNP-environment interactions significant for the disease (in accordance with the GWAS data) were also registered in the studied sample of patients, both men and women (higher values of blood glucose, smokers, hypercholesterolemia, etc.).

Together with this, an important value in the HTN susceptibility is played by sex hormones, which, firstly, are directly involved in the process regulation of vasoconstriction/vasodilation [90,91,92,93]; secondly, they have a pronounced influence on a number of cardiovascular/HTN risk factors (distribution of adipose tissue in the body, the development of obesity and metabolic syndrome, the formation of obesity-dependent/independent insulin resistance, etc.) [7]; thirdly, they can be significant “modifiers” in the phenotypic manifestation of potential genetic determinants of HTN by modulating various nuclear and extra-nuclear pathways that control the expression of multiple genes, post-translational modifications of protein molecules, various HTN-impact signaling pathways, etc. [94,95]. Obviously, estrogens cause a BP decrease in premenopausal women and, accordingly, have a protective effect on HTN development [90,91,92,93]. Estrogens realize their HTN-protective phenotypic effects through vasoconstriction/vasodilation mechanisms due to the regulation of the renin–angiotensin–aldosterone system, the production of catecholamines, endothelins and angiotensin II [91,92,93]. After the menopause, the HTN-protective effects of estrogen in women decrease and the risk of raised BP increases [90]. It is assumed that testosterone, by increasing the activity of the renin–angiotensin–aldosterone system, promotes the development of oxidative stress, leading to the increased production of vasoconstrictors and a decrease in the effects of vasodilators (nitric oxide), which predetermines a higher blood pressure level and, accordingly, a higher HTN risk in men [92]. With age, testosterone levels in men decrease; however, the HTN risk does not decrease as expected, but rather increases due to a decrease in the regulatory effect of testosterone on adipose tissue (such as the suppression of adipocyte proliferation, decreased stromal vascular growth, androgen receptor deficiency, etc.) [7,96]), which, in turn, determines an increased risk of abdominal (visceral) obesity in men [7,97,98]. Thus, an age-dependent decrease in testosterone levels in men increases the risk of visceral obesity and thereby increases the HTN risk [7]. The data on the effect of testosterone on the development of obesity in women are ambiguous; there is evidence of a connection between high testosterone levels and both low visceral fat [99] and high fat content [100].

There is convincing evidence of correlations of sex-specific differences in the functioning of the autonomic nervous system and related features of the immune status/reactions of the body with the HTN risk [90,101]. It has been recorded that, in women compared with men, with age and also with obesity, the activity of the sympathetic nervous system is increased [90]. A change in the activity of the sympathetic nervous system has a direct regulatory effect on T cells, which in turn activates various signaling pathways of innate and adaptive immunity (production of various cytokines of pro-inflammatory action, pro-/anti- inflammatory cytokines signaling, interferon-γ-mediated reactions, activation of natural killer cells and monocytes, vascular inflammation, etc.) [85,87,101,102]. Along with the sympathetic nervous system, sex hormones are also involved in the regulation of the innate/adaptive immune system molecular mechanisms (estrogens have an immunostimulating effect while androgens have an immunosuppressive effect) [95]. Interestingly, the materials derived by us from data bioinformatic analysis indicate the paramount importance of immune mechanisms/processes (such as MHC proteins, antigen processing and presentation, and more than 100 other different immune pathways) in the HTN susceptibility in both men and women.

Our in silico data indicate a connection of HTN with a number of genes that control the organism’s immune responses. These are genes such as *HLA* system (*HLA-DRB6*; *HLA-DRB5*; *HLA-DRB1*; *HLA-DQA1*; *HLA-S*; *HLA-B*), *HSP* (*heat shock protein:HSPA1A*; *HCP5*; *HSPA1B*), *LY6* (*lymphocyte antigen 6:LY6G5C*; *LY6G5B*; *LY6G6C*; *LY6G6E*; *LY6G6D*; *LY6G6F*), etc., the expression/splicing of which is regulated by HTN-associated polymorphisms in various organs, including those that are targets for disease (heart/aorta/coronary and other arteries). It is believed that the HLA background shapes the T cell receptor (TCR) repertoire by neglecting/favoring specific T cell subgroups represented by T cell receptor variable beta chain (TCRBV) usage [84]. Different HLAs present auto-/foreign antigens to TCRBV-specific subpopulations of T cells in different ways (better/worse), which determines the individual specific features of interactions in the HLA–TCR system and is important in the formation of susceptibility to various immuno-significant diseases, including hypertension [84,85]. It seems important to point out the presence of clearly expressed differences between men and women regarding the effect of HLA genes on TCRBV transcripts (CD8-T cells in men affected by autoimmune disorders had the ability to multiply in the absence of TCR expression with similarities in key HLA-binding regions), which are supposed to be based on hormone-mediated mechanisms [84]. HSPs act as regulators of the organism’s immune responses, and they are produced when the body is exposed to various damaging/stressful factors (mechanical/oxidative stress, cytokine influences, etc.) and they are the “protective” response of the cells (including cells of the arterial wall) to these effects [86,87]. T cells, interacting with HSPs (primarily HSP60, HSP70), form a regulatory T-cell response of anti-inflammatory directivity [88]. So, momentary effects of HSPs in HTN are protective due to suppression of the NF-κB pathway and improve the BP reply to angiotensin II [88]. Nonetheless chronic hyperexpression of HSP70 probably has prohypertensive value due to its capacity to evoke autoimmune processes [88]. Data from the literature on the increased formation of some HSPs (HSP70, HSP72) in HTN patients (including arteries (adventitial areas) and kidney) are presented [87]. In the work by Li et al., the relationship of a number of polymorphisms of three *HSP70* genes (*HSPA1A*; *HSPA1B*; *HSPA1L*) both independently and as part of individual haplotypes with HTN, was shown in Uygur [86]. Of importance, the involvement of two of these genes (such as *HSPA1A* and *HSPA1B*) in the disease was also established by our in silico analysis. LY6 protein family members (including those considered in our work; gene products such as *LY6G5C*; *LY6G5B*; *LY6G6C*; *LY6G6E*; *LY6G6D*; *LY6G6F*) interacting with various endogenic regulatory factors (interferon-γ, type I and II interferons, retinoic acid,β2-integrins, matrix metalloproteinases, lymphotoxin alpha, etc.) affect immunity-significant cellular functions (T cell activation/proliferation, CD8+ T cell migration, cell adhesion, B cell specification, neutrophil recruitment, etc.) which are essential for the wide range of HTN-involved processes such as inflammation progression, activity of complement, neuronal activity, angiogenesis, etc. [103]. Despite the presence of certain sex-specific features of the immune pathways involved in HTN biology, as indicated in the literature (women, in comparison with men, are more likely to have inflammatory/autoimmune disorders that increase the HTN risk; have greater numbers of circulating IgM and more CD4 T cells; higher infiltration of the kidneys by T cells with an increase in the number of Th17 cells; an increase in the content of regulatory T cells in adipose with weight gain in women, and a pattern of reverse orientation in men; differences in HLA-mediated T-cell selection/expansion, predestining the beta chain of the determining the features of the beta chain of the T cell receptor, differences in the pro-/anti- inflammatory cytokines signaling, etc.) [84,85,102], according to our bioinformatic data, almost all of the detected HTN-significant immune pathways were the same in men and women (due to the almost identical HTN susceptibility gene list in men [147 genes] and women [159 genes], determined by us on the basis of in silico data).

Importantly, all of the above-described HTN-important environmental risk factors, biological processes/mechanisms (state of the autonomic nervous system, immune status, hormonal background, etc.) are closely correlated with each other and represent a sophisticated multi-level, multi-stage and multi-directional sex-specific system of regulation of BP-related traits (phenotypes) in HTN. There is no doubt that all these factors discussed above can be powerful epigenetic modifiers of the phenotypic manifestation of HTN predisposition genes and determine men–women differences in the involvement of genetic determinants in susceptibility to the disease. This study shows differences in the nature of the HTN genetic determination in men and women both within the framework of the main effects of BP/HTN-associated GWAS SNPs and their intergenic interactions: in women, susceptibility to the disease is determined by the polymorphism *HFE* rs1799945 C > G and strongly pronounced inter-locus interactions of all 10 examined SNPs (26 intergenic interactions were identified models), whereas in men, predisposition to the disease was associated with *BAG6* rs805303 G > A polymorphism and significantly less pronounced interactions between only 8 considered loci (just 7 models were founded).

It is quite interesting that the data of this work are largely consistent with our previously published materials in which, during an associative study of a women sample with pre-eclampsia/without pre-eclampsia from the identical population of Europeans in Central Russia (the list of studied loci was analogous), it was found that the *HFE* rs1799945 C > G increased the pre-eclampsia risk (for allele G OR = 2.24) and *BAG6* rs805303 G > A decreased the pre-eclampsia risk (for allele A OR = 0.55–0.78) [104] including this pregnancy complication risk in women with BMI ≥ 25 (for allele A OR = 0.36–0.66) [105]. So, the polymorphic variant G rs1799945 *HFE* increases both the HTN risk in women (data from this work, OR = 11.15 for genotype GG) and the pre-eclampsia risk in women (a specific symptom of this complication of pregnancy is increased BP (OR= 2.24 [104])), which may indicate the “universality” of this genetic marker as a risk factor for the development of hypertensive conditions in European women in Central Russia, and allows us to recommend its use in practical medicine in order to distinguish women with a high risk of developing elevated BP. Concurrently, there are some discrepancies in the results of these studies: SNP *BAG6* rs805303 G > A has a protective value for HTN in men (not in women!) (OR = 0.30), but in parallel, this polymorphism marks a low risk of developing pre-eclampsia in pregnant women as a whole (OR = 0.55–0.78) [104] and among women with a BMI ≥ 25 (OR = 0.36–0.66) [105]. One of the possible reasons for these sex-specific differences in the value of the *BAG6* rs805303 G > A locus in the development of pathology with elevated BP may be the modifying effect on the phenotypic manifestation of this polymorphism of an unequal confounder factor list taken into account in these studies (BMI, TC, TG, HDL-C, LDL-C, blood glucose, smokers—present study; age, family history of PE, pre-pregnancy BMI, obesity, number of gravidities, spontaneous/induced abortions, stillbirths, smokers—previous study [104]). At the same time, there is an obvious need to continue studies on the association of SNP *BAG6* rs805303 G > A with diseases associated with elevated BP in the studied population, in order to finally establish its predictive potential (including their sex-specific features).

This study has a number of limitations. Firstly, experimental confirmations of the functional effects of HTN-significant GWAS loci identified in silico by us (influence on expression, splicing, epigenetic modifications of genes) are needed. Secondly, it is necessary to confirm (in silico and experimentally) men–women differences in the functional effects of GWAS-significant loci. Thirdly, an increase in the number of samples of men and women under consideration would allow the identification of phenotypic effects (association with the disease) and others which were “weaker” for this population of GWAS loci. Fourthly, the expansion of the panel of studied GWAS loci would allow to expand the data on men–women features of the genetic determination of HTN. It should be noted that conducting similar studies in other ethno-territorial groups of the population can apparently “show” other patterns (different from our results) since these studies (as well as our work) will be replicative and their results will be largely determined by various features (the structure of the gene pool and the associated features of the “main” effects of genes and the nature of intergenic interactions, the spectrum and severity of environmental risk factors and the associated features of gene–environmental interactions, etc.) of those ethno-territorial groups of the population that will be studied.

## 4. Materials and Methods

### 4.1. Study Subjects

Two groups of European subjects of Russia (*n* = 1405 in total, all participants were born in Central Russia and have Russian origin (self-reported) [106,107], such as men (*n* = 821 in total: *n* = 564 HTN, *n* = 257 control) and women (*n* = 584 in total: *n* = 375 HTN, *n* = 209 control) were included in this “case-control” association study. The clinical examination of participants was performed during the 2013–2016 period in the “cardiology department” at the “St. Joasaph Belgorod Regional Clinical Hospital”. Diagnosis of HTN (or absence of HTN) was carried out by qualified cardiologists according to the standards set out in the WHO methodological guidelines [1] (information about this has been presented previously [42]). BP indicators were confirmed by Korotkov (auscultative method using a sphygmomanometer) [108]. BP was measured at least twice within a few days. Thirty minutes before the procedure, the subjects did not consume caffeine/smoke/exercise. The measurement was carried out in the patient’s sitting position after a five-minute rest. BP was measured on both hands; at least two measurements were taken with an interval of one to two minutes between measurements. As an indicator of an individual’s BP, the average value for two measurements taken at least twice was taken. The HTN group was formed from the clinic’s (cardiology department) patients. All HTN patients had a clinical history of disorder for one year or more and 81.79% (82.80% men and 80.27% women) received antihypertensive drugs. The absence of HTN (parameters BP were lower than 140mmHg for SBP and lower than 90mmHg for DBP), coronary artery disease and type 2 diabetes mellitus were the basis for inclusion in the control group. Persons who do not suffer from HTN (control group) were recruited during regular (annual) medical examinations at the aforementioned clinical hospital (these examinations were carried out by doctors of various specialties, including qualified cardiologists). All of the participants (HTN and HTN-free) did not have severe chronic allergic/autoimmune/hematological/oncological pathology [109]. Blood specimens of the subjects for defining TC, TG, LDL-C, HDL-C and blood glucose were obtained in the morning (7–9 h) after an eight-hour fast. The implementation of this study was supervised by the Ethics Committee (Human Investigation Committee) at “Belgorod State University” and was accompanied by the written consent of all the subjects.

Information about lifestyle and diet was collected for each subject (patient/control). The consumption of vegetables and fruits in an amount of less than 400 g daily (excluding salted/pickled vegetables, and starchy vegetables (potatoes)) was considered “low fruit/vegetable consumption” [110]. The average weekly physical activity at work and at home related to transport and recreation (including walking, running, fitness club classes, etc.) less than 150 min of moderate-intensity physical activity (for example, brisk walking) for 30 min or longer, at least five times a week) was considered “low physical activity”. [111,112]. The average daily intake of fatty foods of more than 10% of the total food consumed (daily energy consumption due to fatty foods in the total amount of daily energy) was considered “high fatty food consumption” [110]. Daily salt intake (sodium chloride) of 5 g (teaspoon) or more per day was considered “high sodium consumption” [110].

### 4.2. Laboratory DNA Testing

We gathered peripheral blood (leukocytes) to extract genomic DNA [113] (the phenol/chloroform DNA extraction methodology was presented earlier [114]). The ten specially selected polymorphisms (confirmed associations of GWAS level with blood pressure (BP) parameters and/or HTN in European (Table S17) and assumed functional ability [104,105,115] (HaploReg information was regarded [116]) (Table S18)) were considered. The list of studied loci was as follows: (*PLCE1*) rs932764 A > G, (*AC026703.1*) rs1173771 G > A, (*CERS5*) rs7302981 G > A, (*HFE*) rs1799945 C > G, (*OBFC1*) rs4387287 C > A, (*BAG6*) rs805303 G > A, (*RGL3*) rs167479 T > G, (*ARHGAP42*) rs633185 C > G, (*TBX2*) rs8068318 T > C and (*ATP2B1*) rs2681472 A > G. All ten loci were correlated with BP in Europeans and all ten SNPs were associated with HTN: eight SNPs were HTN-linked in Europeans and two loci (rs4387287 *OBFC1* and rs2681472 *ATP2B1*) were disorder-associated in the sample with a predominance (>85%) of Europeans (Table S17). Nine loci out of ten (excluding rs4387287 *OBFC1*) were associated with HTN/BP in two or more GWAS (Table S17). All selected ten SNPs have significant functionality (Table S18). One of the generally accepted methods of genotyping (the allelic discrimination method) and the CFX96 RT System device (Bio-Rad Laboratories, Hercules, CA, USA) were used for laboratory genetic studies [117]. The participants case/control status was masked throughout the laboratory genetic analysis. Genotyping of a random duplicated sample (near 4–6% from all sample) was utilized as an independent internal control to provide the individual genotyping data quality assurance [118,119]. No genotyping errors were registered.

### 4.3. Association Statistical Analysis

The genotypes of the examined 10 loci were tested for H–W equilibrium [120,121]. The sex-specific disorder-impact genetic association of individual polymorphisms [additive/recessive/dominant/allelic common model was calculated [122]] and interaction between SNPs [123] and HTN was appreciated based on the results (OR with 95%CI was evaluated [124,125]) obtained in the gPLINK [126], MDR [127,128], MB-MDR [129,130] genetic programs. In the logistic regression calculations, we took into account covariates (BMI, TC, TG, LDL-C, HDL-C, blood glucose, smokers (Model 1) and BMI, TC, TG, LDL-C, HDL-C, blood glucose, smokers, low physical activity, high fatty food consumption (Model 2) in both men and women (Table 1)), performed permutation procedures (in order to minimize the probability of false positive results [131,132]) and the results’ analysis was carried out on the basis of a stronger level of statistical significance, p_perm-Bonferroni_ ≤ 0.025 (Bonferroni’s correction related to the amount of comparison pairs studied was accounted, *n* = 2 [men and women]). For individual SNPs statistical power was estimated by Quanto (v.1.2.4) [133].

### 4.4. Definition of the Alleged Functional Ability of HTN-Related Polymorphisms and Genes

For the purpose of biological interpretation of the identified associations (establishing the mechanisms underlying these associations), we used in silico information on the assumed functional ability of HTN-related polymorphisms (taking into account strongly coupled loci with a coupling strength of at least 0.80 [134,135]) and genes. We used such seven bioinformatics databases as (1) HaploReg [116] (determination of the regulatory potential of polymorphisms: location in putative promoters/enhancers, association with transcription factors/regulatory proteins, localization in regions of open chromatin and evolutionarily conservative DNA sites), (2) SIFT [136] and (3) PolyPhen-2 [137] (identification and evaluation of predictive potential of non-synonymous SNPs), (4) GTEx [138] (correlation of loci with gene expression and alternative splicing in 54 different organs/tissues), (5) Blood eQTL browser [139] (the relationship of SNPs with gene expression in peripheral blood), (6) Gene Ontology [140] (identification of HTN-associated genes pathways), (7) GeneMANIA [141] (estimation and visualization of the mechanisms of intergenic interactions of HTN-significant genes).

## 5. Conclusions

This study showed a more strongly pronounced contribution of BP/HTN-associated GWAS SNPs to HTN susceptibility (due to weightier intergenic interactions) in European women than in men.

## Figures and Tables

**Table 1:** Phenotypic characteristics of the study participants.

Parameters	Men (*n* = 821)			Women (*n* = 584)		
	HTN, Mean ± SD, % (*n*)	Controls, Mean ± SD, % (*n*)	*p*	HTN, Mean ± SD, % (*n*)	Controls, Mean ± SD, % (*n*)	*p*
N	564	257	-	375	209	-
Age (years)	57.60 ± 8.36	57.54 ± 9.73	0.86	58.80 ± 9.64	58.17 ± 9.30	0.43
BMI (kg/m^2^)	30.76 ± 4.52	25.04 ± 2.86	<0.001	30.81 ± 5.84	24.83 ± 3.41	<0.001
SBP (mmHg)	180.45 ± 27.40	123.89 ± 11.92	<0.001	185.53 ± 29.26	120.98 ± 10.76	<0.001
DBP (mmHg)	104.76 ± 12.75	78.19 ± 6.76	<0.001	107.47 ± 14.35	76.98 ± 7.09	<0.001
TC (mM)	5.65 ± 1.32	5.24 ± 0.98	<0.001	5.79 ± 1.23	5.28 ± 1.09	<0.001
HDL-C (mM)	1.35 ± 0.44	1.48 ± 0.41	<0.001	1.33 ± 0.37	1.56 ± 0.42	<0.001
LDL-C (mM)	3.78 ± 0.73	3.18 ± 0.66	<0.001	3.77 ± 1.15	3.25 ± 0.79	<0.001
TG (mM)	2.00 ± 1.01	1.28 ± 0.81	<0.001	1.80 ± 1.04	1.18 ± 0.62	<0.001
Blood glucose (mM)	5.82 ± 1.20	4.93 ± 1.12	<0.001	6.08 ± 1.91	4.81 ± 0.67	<0.001
Smoking	65.58 (318)	34.96 (74)	<0.001	9.75 (35)	4.78 (10)	0.04
Alcohol abuse	9.30 (52)	2.29 (13)	0.17	0.28 (1)	0 (0)	1.00
Antihypertensive medication use	82.80 (467)	-	-	80.27 (301)	-	-
HTN Grade: Grade 1	18.44 (104)	-	-	12.80 (48)	-	-
Grade 2	48.23 (272)	-	-	45.60 (171)	-	-
Grade 3	33.33 (188)	-	-	41.60 (156)	-	-
HTN Stage: Stage 1	11.88 (67)	-	-	7.20 (27)	-	-
Stage 2	31.21 (176)	-	-	30.40 (114)	-	-
Stage 3	56.91 (321)	-	-	62.40 (234)	-	-
Stroke incident	35.64 (201)	-	-	27.20 (102)	-	-
Coronary artery disease	29.08 (164)	-	-	32.00 (120)	-	-
Type 2 diabetes mellitus	33.51 (189)	-	-	30.13 (113)	-	-
Low physical activity	57.62 (325)	24.12 (62)	<0.001	60.27 (226)	31.58 (66)	<0.001
Low fruit/vegetable consumption	11.52 (65)	7.39 (19)	0.09	11.20 (42)	6.70 (14)	0.10
High fatty food consumption	24.47 (138)	10.12 (26)	<0.001	25.06 (94)	10.53 (22)	<0.001
High sodium consumption	16.49 (93)	14.01 (36)	0.42	17.07 (64)	12.44 (26)	0.17

Note: Clinical characteristics of age, BMI, SBP, DBP, HDL-C, LDL-C, TG and TC are given as means ± SD and other values as number of individuals; BMI—body mass index; SBP—systolic blood pressure; DBP—diastolic blood pressure; TC—total cholesterol; HDL-C—high-density lipoprotein cholesterol; TG—triglycerides; LDL-C—low-density lipoprotein cholesterol; HTN Grade and Stage are presented according to [1]: Grade 1 SBP = 140–159 mmHg and/or DBP = 90–99 mmHg; Grade 2 SBP = 160–179 mmHg and/or DBP = 100–109 mmHg; Grade 3 SBP > 180 mmHg and/or DBP > 110 mmHg).

**Table 2:** Associations of the studied gene polymorphisms with HTN in men and women.

Gene (SNP, Major/Minor Alleles)	*n*	Allelic Model				Additive Model				Dominant Model				Recessive Model			
		OR	95%CI		*p*	OR	95%CI		*p*	OR	95%CI		*p*	OR	95%CI		*p*
			L95	U95			L95	U95			L95	U95			L95	U95	
**Model 1**																	
Men																	
*AC026703.1* (rs1173771, G/A)	774	0.91	0.73	1.13	0.383	0.88	0.61	1.29	0.524	0.67	0.38	1.17	0.161	1.25	0.61	2.55	0.542
*HFE* (rs1799945, C/G)	800	1.16	0.89	1.52	0.281	1.30	0.85	1.99	0.220	1.38	0.80	2.37	0.248	1.54	0.54	4.38	0.421
*BAG6* (rs805303, G/A)	789	0.95	0.76	1.19	0.665	0.66	0.46	0.96	0.028	0.82	0.49	1.39	0.466	**0.30**	**0.15**	**0.61**	**0.0008**
*PLCE1* (rs932764, A/G)	775	1.23	0.99	1.53	0.056	1.24	0.85	1.80	0.267	1.30	0.71	2.36	0.392	1.36	0.72	2.55	0.339
*OBFC1* (rs4387287, C/A)	751	0.88	0.66	1.17	0.380	0.84	0.50	1.41	0.497	0.80	0.44	1.46	0.469	0.88	0.17	4.72	0.884
*ARHGAP42* (rs633185, C/G)	800	1.01	0.79	1.28	0.964	1.18	0.80	1.74	0.418	1.14	0.68	1.90	0.631	1.58	0.65	3.86	0.316
*CERS5* (rs7302981, G/A)	767	0.93	0.74	1.16	0.499	0.67	0.45	0.99	0.044	0.70	0.40	1.22	0.211	0.44	0.21	0.92	0.028
*ATP2B1* (rs2681472, A/G)	778	1.12	0.82	1.54	0.479	1.64	0.91	2.97	0.103	1.61	0.83	3.09	0.157	5.61	0.42	74.38	0.191
*TBX2* (rs8068318, T/C)	762	1.14	0.89	1.46	0.299	1.33	0.87	2.05	0.194	1.45	0.85	2.49	0.173	1.33	0.46	3.83	0.596
*RGL3* (rs167479, T/G)	783	0.98	0.79	1.21	0.833	0.82	0.57	1.18	0.283	0.90	0.49	1.64	0.722	0.66	0.37	1.18	0.161
Women																	
*AC026703.1* (rs1173771, G/A)	543	0.91	0.71	1.17	0.458	0.92	0.64	1.31	0.631	0.85	0.50	1.45	0.554	0.95	0.52	1.76	0.877
*HFE* (rs1799945, C/G)	573	0.71	0.53	0.96	0.026	0.75	0.48	1.17	0.210	0.60	0.37	0.98	0.040	**11.15**	**1.15**	**97.68**	**0.011**
*BAG6* (rs805303, G/A)	560	0.99	0.77	1.28	0.938	1.08	0.76	1.51	0.675	1.06	0.66	1.72	0.801	1.18	0.60	2.35	0.630
*PLCE1* (rs932764, A/G)	544	0.95	0.74	1.21	0.663	0.83	0.59	1.17	0.286	0.59	0.34	1.03	0.061	1.07	0.59	1.91	0.832
*OBFC1* (rs4387287, C/A)	509	0.97	0.72	1.30	0.891	0.88	0.57	1.35	0.551	0.89	0.52	1.50	0.650	0.70	0.21	2.25	0.545
*ARHGAP42* (rs633185, C/G)	577	1.04	0.79	1.37	0.759	1.03	0.71	1.49	0.868	1.05	0.65	1.68	0.850	1.02	0.42	2.46	0.965
*CERS5* (rs7302981, G/A)	535	1.20	0.93	1.55	0.152	1.19	0.84	1.68	0.326	1.03	0.79	2.15	0.306	1.20	0.62	2.30	0.591
*ATP2B1* (rs2681472, A/G)	551	0.96	0.68	1.35	0.809	0.89	0.55	1.45	0.648	0.94	0.55	1.59	0.808	0.45	0.08	2.62	0.372
*TBX2* (rs8068318, T/C)	530	1.06	0.80	1.40	0.704	1.04	0.70	1.55	0.841	1.03	0.63	1.68	0.918	1.16	0.42	3.18	0.772
*RGL3* (rs167479, T/G)	550	1.15	0.90	1.47	0.266	1.29	0.92	1.82	0.138	1.44	0.83	2.50	0.200	1.38	0.79	2.41	0.252
**Model 2** *																	
Men																	
*AC026703.1* (rs1173771, G/A)						0.90	0.62	1.30	0.560	0.68	0.39	1.19	0.176	1.25	0.62	2.51	0.529
*HFE* (rs1799945, C/G)						1.33	0.87	2.01	0.187	1.42	0.83	2.43	0.204	1.55	0.56	4.29	0.403
*BAG6* (rs805303, G/A)						0.66	0.46	0.95	0.026	0.80	0.47	1.34	0.395	**0.31**	**0.16**	**0.64**	**0.001**
*PLCE1* (rs932764, A/G)						1.21	0.84	1.75	0.311	1.27	0.71	2.29	0.425	1.31	0.71	2.44	0.391
*OBFC1* (rs4387287, C/A)						0.90	0.54	1.50	0.681	0.86	0.48	1.56	0.629	1.03	0.19	5.64	0.974
*ARHGAP42* (rs633185, C/G)						1.14	0.77	1.67	0.510	1.10	0.66	1.83	0.727	1.49	0.62	3.76	0.376
*CERS5* (rs7302981, G/A)						0.67	0.46	1.00	0.047	0.70	0.40	1.22	0.213	0.46	0.22	0.94	0.033
*ATP2B1* (rs2681472, A/G)						1.59	0.89	2.83	0.118	1.55	0.82	2.95	0.179	5.11	0.42	61.98	0.200
*TBX2* (rs8068318, T/C)						1.29	0.85	1.97	0.233	1.40	0.82	2.38	0.220	1.33	0.48	3.75	0.584
*RGL3* (rs167479, T/G)						0.80	0.56	1.15	0.237	0.90	0.49	1.65	0.740	0.63	0.35	1.11	0.110
Women																	
*AC026703.1* (rs1173771, G/A)						0.92	0.65	1.31	0.649	0.87	0.51	1.47	0.595	0.95	0.51	1.74	0.858
*HFE* (rs1799945, C/G)						0.77	0.50	1.19	0.241	0.61	0.37	0.99	0.043	**10.96**	**1.12**	**89.68**	**0.0** **12**
*BAG6* (rs805303, G/A)						1.05	0.75	1.47	0.781	1.05	0.65	1.69	0.848	1.10	0.56	2.19	0.776
*PLCE1* (rs932764, A/G)						0.80	0.57	1.13	0.211	0.58	0.33	1.00	0.050	1.00	0.56	1.77	0.997
*OBFC1* (rs4387287, C/A)						0.89	0.58	1.37	0.593	0.91	0.54	1.54	0.726	0.67	0.21	2.16	0.502
*ARHGAP42* (rs633185, C/G)						1.02	0.71	1.48	0.898	1.03	0.65	1.65	0.895	1.03	0.43	2.47	0.951
*CERS5* (rs7302981, G/A)						1.20	0.85	1.68	0.305	1.34	0.81	2.21	0.250	1.16	0.61	2.21	0.649
*ATP2B1* (rs2681472, A/G)						0.98	0.61	1.56	0.920	1.00	0.60	1.68	0.989	0.70	0.13	3.78	0.678
*TBX2* (rs8068318, T/C)						1.07	0.72	1.58	0.754	1.03	0.63	1.68	0.899	1.31	0.48	3.54	0.602
*RGL3* (rs167479, T/G)						1.20	0.86	1.68	0.274	1.32	0.77	2.27	0.312	1.25	0.72	2.16	0.435

Note: * For Model 2, calculations of the allelic model were not performed because their results are identical to those of Model 1 (covariates are not used when calculating the allelic model); all results were obtained after adjustment for covariates; list covariates for Model 1: BMI, TC, TG, LDL-C, HDL-C, blood glucose, smokers; list covariates for Model 2: BMI, TC, TG, LDL-C, HDL-C, blood glucose, smokers, low physical activity, high fatty food consumption; OR—odds ratio; 95% CI-95% confidence interval; *p* values ≤ 0.025 are shown in bold.

**Table 3:** SNP × SNP interactions significantly associated with HTN in men and women.

N	SNP × SNP Interaction Models	NH	*beta*H	WH	NL	*beta*L	WL	p_perm_
**Men**								
Three-order interaction models								
1	rs805303 *BAG6* × rs1173771 *AC026703.1* × rs4387287 *OBFC1*	1	2.287	4.73	4	−0.815	16.76	0.008
2	rs932764 *PLCE1* × rs7302981 *CERS5* × rs1799945 *HFE*	-	-	-	3	−1.118	18.67	0.010
3	rs7302981 *CERS5* × rs1173771 *AC026703.1* × rs167479 *RGL3*	-	-	-	4	−0.933	18.13	0.016
Four-order interaction models								
1	rs932764 *PLCE1* × rs7302981 *CERS5* × rs1799945 *HFE* × rs8068318 *TBX2*	1	2.096	3.75	5	−1.325	32.12	0.001
2	rs932764 *PLCE1* × rs7302981 *CERS5* × rs1799945 *HFE* × rs1173771 *AC026703.1*	0	-	-	5	−1.637	28.51	0.008
3	rs932764 *PLCE1* × rs8068318 *TBX2* × rs805303 *BAG6* × rs1173771 *AC026703.1*	0	-	-	6	−1.265	27.17	0.016
4	rs7302981 *CERS5* × rs1799945 *HFE* × rs805303 *BAG6* × rs167479 *RGL3*	1	0.531	3.07	5	−1.261	24.40	0.022
**Women**								
Two-order interaction models								
1	rs8068318 *TBX2* × rs167479 *RGL3*	3	0.623	10.18	3	−0.880	21.92	<0.001
2	rs932764 *PLCE1* × rs1799945 *HFE*	2	-	-	2	−0.801	10.83	0.013
Three-order interaction models								
1	rs1799945 *HFE* × rs8068318 *TBX2* × rs167479 *RGL3*	2	1.140	21.30	4	−0.898	20.87	<0.001
2	rs2681472 *ATP2B1* × rs8068318 *TBX2* × rs167479 *RGL3*	2	0.804	13.59	2	−0.870	18.15	<0.001
3	rs932764 *PLCE1* × rs7302981 *CERS5* × rs805303 *BAG6*	2	1.011	8.23	2	−1.311	20.96	0.001
4	rs932764 *PLCE1* × rs805303 *BAG6* × rs4387287 *OBFC1*	4	1.394	20.28	1	−1.419	3.43	0.001
5	rs8068318 *TBX2* × rs4387287 *OBFC1* × rs167479 *RGL3*	3	0.778	15.03	4	−0.817	18.36	0.001
6	rs2681472 *ATP2B1* × rs805303 *BAG6* × rs1173771 *AC026703.1*	1	1.404	7.81	3	−1.573	16.48	0.001
7	rs932764 *PLCE1* × rs8068318 *TBX2* × rs167479 *RGL3*	2	1.323	10.47	2	−0.966	18.01	0.001
8	rs932764 *PLCE1* × rs1799945 *HFE* × rs805303 *BAG6*	0	-	-	3	−1.218	17.93	0.001
9	rs932764 *PLCE1* × rs1799945 *HFE* × rs8068318 *TBX2*	1	0.679	8.01	3	−0.849	15.49	0.010
10	rs932764 *PLCE1* × rs1799945 *HFE* × rs167479 *RGL3*	0	-	-	3	−1.102	15.68	0.016
Four-order interaction models								
1	rs932764 *PLCE1* × rs8068318 *TBX2* × rs1173771 *AC026703.1* × rs167479 *RGL3*	1	1.446	3.33	7	−1.275	33.53	<0.001
2	rs1799945 *HFE* × rs8068318 *TBX2* × rs805303 *BAG6* × rs167479 *RGL3*	4	1.045	18.51	7	−1.229	32.64	<0.001
3	rs932764 *PLCE1* × rs1799945 *HFE* × rs1173771 *AC026703.1* × rs167479 *RGL3*	3	1.182	9.42	6	−1.840	31.40	<0.001
4	rs2681472 *ATP2B1* × rs1799945 *HFE* × rs8068318 *TBX2* × rs167479 *RGL3*	4	1.235	27.98	6	−1.276	31.20	<0.001
5	rs1799945 *HFE* × rs8068318 *TBX2* × rs1173771 *AC026703.1* × rs167479 *RGL3*	3	0.995	14.27	7	−1.356	30.93	<0.001
6	rs932764 *PLCE1* × rs1799945 *HFE* × rs8068318 *TBX2* × rs167479 *RGL3*	3	1.256	17.70	5	−1.287	29.85	<0.001
7	rs1799945 *HFE* × rs8068318 *TBX2* × rs4387287 *OBFC1* × rs167479 *RGL3*	3	1.410	26.21	2	−0.707	7.43	<0.001
8	rs2681472 *ATP2B1* × rs633185 *ARHGAP42* × rs7302981 *CERS5* × rs805303 *BAG6*	0	-	-	5	−1.199	26.05	<0.001
9	rs2681472 *ATP2B1* × rs633185 *ARHGAP42* × rs8068318 *TBX2* × rs167479 *RGL3*	2	0.813	9.37	4	−1.192	24.91	<0.001
10	rs2681472 *ATP2B1* × rs8068318 *TBX2* × rs1173771 *AC026703.1* × rs167479 *RGL3*	1	1.157	4.12	4	−1.207	24.60	<0.001
11	rs633185 *ARHGAP42* × rs932764 *PLCE1* × rs7302981 *CERS5* × rs805303 *BAG6*	2	1.503	7.55	2	−1.238	25.86	0.001
12	rs932764 *PLCE1* × rs7302981 *CERS5* × rs1799945 *HFE* × rs167479 *RGL3*	0	-	-	5	−1.477	25.01	0.001
13	rs7302981 *CERS5* × rs1799945 *HFE* × rs8068318 *TBX2* × rs167479 *RGL3*	3	0.995	14.27	5	−1.242	24.28	0.001
14	rs633185 *ARHGAP42* × rs7302981 *CERS5* × rs8068318 *TBX2* × rs167479 *RGL3*	1	0.943	3.36	4	−1.376	24.37	0.010

Note: The results were obtained using the MB-MDR method with adjustment for covariates (Model 1); NH—number of significant high-risk genotypes in the interaction; beta H—regression coefficient for high-risk exposition in the step 2 analysis; WH—Wald statistic for high-risk category; NL—number of significant low-risk genotypes in the interaction; beta L—regression coefficient for low-risk exposition in the step 2 analysis; WL—Wald statistic for low-risk category; p_perm_—permutation *p*-value for the interaction model (1.000 permutations).

## Data Availability

The data generated in the present study are available from the corresponding author upon reasonable request.

## Supplementary Materials

The supporting information can be downloaded at: https://www.mdpi.com/article/10.3390/ijms24097799/s1.
