# Supplementary material for: Sex-Specific Features of the Correlation between GWAS-Noticeable Polymorphisms and Hypertension in Europeans of Russia
Source: Int J Mol Sci. 2023 Apr 25;24(9):7799. doi: 10.3390/ijms24097799 (PMC10178435; doi:10.3390/ijms24097799)
Supplement: Supplementary file 1 [file ijms-24-07799-s001.zip › --Suppl table S1.pdf]

Supplementary table S1. The allele and genotype frequencies of the studied SNPs in the HTN and control groups of male.

Gene (SNP, major/minor alleles)	Minor allele frequency,%	Genotype distribution*	H_o	H_e	P_{HWE}
HTN patients (n=564)					
AC026703.1 (rs1173771,G/A)	41.12	88/259/182	0.490	0.484	0.858
HFE (rs1799945,C/G)	20.86	32/165/352	0.301	0.330	0.039
BAG6 (rs805303,G/A)	33.30	66/229/247	0.423	0.444	0.248
PLCE1 (rs932764,A/G)	50.94	130/280/120	0.528	0.500	0.224
OBFC1 (rs4387287,C/A)	16.80	19/135/361	0.262	0.280	0.157
ARHGAP42 (rs633185,C/G)	26.91	44/208/298	0.378	0.393	0.386
CERS5 (rs7302981,G/A)	38.02	72/256/198	0.487	0.471	0.517
ATP2B1 (rs2681472,A/G)	13.98	15/119/399	0.223	0.241	0.104
TBX2 (rs8068318,T/C)	27.11	45/193/284	0.370	0.395	0.150
RGL3 (rs167479,T/G)	49.63	132/270/136	0.502	0.500	1.000
Control group (n=257)					
AC026703.1 (rs1173771,G/A)	43.47	45/123/77	0.502	0.492	0.796
HFE (rs1799945,C/G)	18.53	11/71/169	0.283	0.302	0.300
BAG6 (rs805303,G/A)	34.41	36/98/113	0.397	0.451	0.066
PLCE1 (rs932764,A/G)	45.71	56/112/77	0.457	0.496	0.246
OBFC1 (rs4387287,C/A)	18.64	6/76/154	0.322	0.303	0.518
ARHGAP42 (rs633185,C/G)	26.80	20/94/136	0.376	0.392	0.520
CERS5 (rs7302981,G/A)	39.83	39/114/88	0.473	0.479	0.893
ATP2B1 (rs2681472,A/G)	12.65	5/52/188	0.212	0.221	0.559
TBX2 (rs8068318,T/C)	24.58	19/80/141	0.333	0.371	0.119
RGL3 (rs167479,T/G)	50.20	65/116/64	0.474	0.500	0.443

Note: * minor allele homozygotes / heterozygotes / major allele homozygotes
