# Supplementary material for: Sex-Specific Features of the Correlation between GWAS-Noticeable Polymorphisms and Hypertension in Europeans of Russia
Source: Int J Mol Sci. 2023 Apr 25;24(9):7799. doi: 10.3390/ijms24097799 (PMC10178435; doi:10.3390/ijms24097799)
Supplement: Supplementary file 1 [file ijms-24-07799-s001.zip › --Suppl Table S17.pdf]

Supplementary table S17. The GWAS data about associations of the studied candidate genes polymorphisms with blood pressure/hypertension, some cardiovascular diseases and anthropometric characteristics.

SNP of gene (position (hg38))	Phenotype	Association (significance) (affected allele)	Reference
rs1173771 *AC026703.1* (5: 32814922)	SBP	β = 0.50 (p = 1.8×10^{-16}) (G)	International Consortium et al., 2011
	DBP	β = 0.26 (p = 9.1×10^{-12}) (G)	
	Hypertension	β = 0.06 (p = 3.2×10^{-10}) (G)	
	MAP	β = 0.28 (p = 3.51×10^{-9}) (G)	Wain L. V., et al., 2011
	PP	β = 0.28 (p = 4.56×10^{-9}) (G)	
	MAP	β = −0.35 (p = 3.19×10^{-8}) (A)	Kato N., et al., 2015
	Hip circumference (sex-combined)	β = 0.03 (p = 6.13×10^{-13}) (A)	Shungin D., et al., 2015
	Hip circumference (men)	β = 0.03 (p = 6.19×10^{-8}) (A)	
	SBP	β = −0.43 (p = 2.0×10^{-28}) (A)	Hoffmann T. J. et al., 2017
	PP	β = −0.25 (p = 1.0×10^{-21}) (A)	
	DBP	β = −0.19 (p = 8.0×10^{-16}) (A)	
	Height	β = 0.04 (p = 9.0×10^{-8}) (A)	Tachmazidou I., et al., 2017
	SBP	β = 0.51(p = 3.20×10^{-12}) (G)	Wain L. V., et al., 2017
	PP	β = 0.28 (p = 2.36×10^{-9}) (G)	
	SBP (smoking interaction) (European)	p = 1.74×10^{-38}	Sung Y. J., et al., 2018
	DBP (smoking interaction) (European)	p = 3.62×10^{-22}	
	SBP (smoking interaction) (trans-ethnic data)	p = 8.25×10^{-44}	
	DBP (smoking interaction) (trans-ethnic data)	p = 2.75×10^{-28}	
	SBP	β = −0.42 (p = 4.19×10^{-8}) (A)	Takeuchi F., et al., 2018
	MAP	β = −0.29 (p = 5.39×10^{-8}) (A)	
rs1799945 *HFE* (6: 26090951)	DBP	β = 0.46 (p = 1.5×10^{-15}) (G)	International Consortium et al., 2011
	SBP	β = 0.62 (p = 7.7×10^{-12}) (G)	
	Hypertension	β = 0.10 (p = 1.8×10^{-10}) (G)	
	DBP	β = −0.43 (p = 3.1×10^{-16}) (C)	Ehret G. B. et al., 2016
	SBP	β = −0.60 (p = 3.28×10^{-12}) (C)	
	DBP	β = −0.29 (p = 1.0×10^{-18}) (C)	Hoffmann T. J. et al., 2017
	SBP	β = −0.36 (p = 4.0×10^{-11}) (C)	

	DBP	β =0.52 (p = 9.2×10^{-18}) (G)	Liu C. et al., 2016
	MAP	β = 0.58 (p = 1.0×10^{-17}) (G)	
	SBP	β = 0.70 (p = 6.2×10^{-13}) (G)	
	Hypertension	Z score = 0.039 (p = 3.7×10^{-9}) (G)	
	DBP (trans-ethnic data)	β = −0.03 (p = 1.26×10^{-19}) (C)	Surendran P. et al., 2016
	DBP (European)	β = −0.03 (p = 9.82×10^{-10}) (C)	
	DBP	β = 0.47 (p = 8.87×10^{-14}) (G)	Wain L. V. et al., 2017
	SBP	β = 0.63 (p = 7.63×10^{-10}) (G)	
	DBP (smoking interaction) (European)	p = 1.51×10^{-31}	Sung Y. J. et al., 2018
	SBP (smoking interaction) (European)	p = 8.46×10^{-17}	
	DBP (smoking interaction) (trans-ethnic data)	p = 3.77×10^{-33}	
	SBP (smoking interaction) (trans-ethnic data)	p = 7.43×10^{-19}	
rs805303 *BAG6/ BAT2-BAT5* (6: 31648589)	SBP	β = 0.37 (p = 1.5×10^{-11}) (G)	International Consortium et al., 2011
	DBP	β = 0.23 (p = 3.0×10^{-11}) (G)	
	Hypertension	β = 0.05 (p = 1.1×10^{-10}) (G)	
	SBP (European)	β = −0.02 (p = 3.02×10^{-6}) (A)	Surendran P. et al., 2016
rs932764 *PLCE1* (10: 94136183)	SBP	β = 0.48 (p = 7.1×10^{-16}) (G)	International Consortium et al., 2011
	Hypertension	β = 0.06 (p = 9.4×10^{-9}) (G)	
	SBP	β = −0.50 (p = 6.88×10^{-17}) (A)	Ehret G. B., et al., 2016
	DBP	β = −0.22 (p = 6.28×10^{-10}) (A)	
	SBP	β = −0.30 (p = 1.0×10^{-14}) (A)	Hoffmann T. J. et al., 2016
	PP	β = −0.16 (p = 3.0×10^{-10}) (A)	
	PP	β = 0.26 (p = 5.67×10^{-8}) (G)	Wain L. V., et al., 2017
rs4387287 *OBFC1* (10:103918139)	DBP (European)	β= 0.22 (p=5.55 x 10^{-8}) (A)	Surendran P. et al., 2016
	SBP (European)	β= 0.34 (p=2.21 x 10^{-7}) (A)	
	DBP (trans-ethnic data)	β= 0.22 (p=4.21 x 10^{-10}) (A)	
	SBP (trans-ethnic data)	β= 0.36 (p=9.12 x 10^{-10}) (A)	
	Hypertension (trans-ethnic data)	Z score = 5.58 (p = 2.37 x 10^{-8}) (A)	
rs633185 *ARHGAP42* (11:100722807)	SBP	β = −0.57(p = 1.2×10^{-17}) (G)	International Consortium et al., 2011
	DBP	β = −0.33(p = 2.0×10^{-15}) (G)	
	Hypertension	β = −0.07(p = 6.4×10^{-11}) (G)	

	SBP	β = 0.52 (p = 6.97×10^{-15}) (C)	Ehret G. B., et al., 2016
	DBP	β = 0.29 (p = 2.38×10^{-12}) (C)	
	SBP	β = −0.49 (p = 1.0×10^{-31}) (G)	Hoffmann T. J. et al., 2016
	DBP	β = −0.27 (p = 1.0×10^{-26}) (G)	
	PP	β = −0.22 (p = 1.0×10^{-15}) (G)	
	SBP	β = 0.52 (p = 8.43×10^{-11}) (C)	Wain L. V., et al., 2017
	DBP	β = 0.27 (p = 2.33×10^{-8}) (C)	
	SBP x alcohol consumption interaction	β = 0.54 (p = 2.24×10^{-29}) (C)	Feitosa M. F. et al., 2018
	MAP x alcohol consumption interaction	β = 0.22 (p = 2×10^{-12}) (C)	
	MAP	β = 0.39 (p = 2.56×10^{-13}) (C)	Takeuchi F., et al., 2018
	SBP	β = 0.51 (p = 7.16×10^{-12}) (C)	
	DBP	β = 0.33 (p = 8.93×10^{-12}) (C)	
	Hypertension	β = 0.08 (p = 5.13×10^{-10}) (C)	
	Coronary artery disease	(p = 8.81×10^{-9}) (C)	Zhou W., et al., 2018
	High blood pressure and chronic obstructive pulmonary disease	(p = 1.18×10^{-47})	Zhu Z., et al., 2019
	SBP (smoking interaction) (European)	p = 8.44×10^{-30}	Sung Y. J., et al., 2018
	DBP (smoking interaction) (European)	p = 2.68×10^{-30}	
	SBP (smoking interaction) (trans-ethnic data)	p = 1.80×10^{-40}	
	DBP (smoking interaction) (trans-ethnic data)	p = 1.18×10^{-40}	
	MAP	β = 0.03 (p = 3.05×10^{-50}) (C)	Sakaue S. et al., 2021
rs7302981 *CERS5/AC074032.1* (12:50144032)	Hypertension	Z score = 6.23 (p= 4.8 x 10^{-10}) (A)	Liu C. et al., 2016
	SBP	β = 0.37 (p=9.4 x 10^{-15}) (A)	
	DBP	β = 0.25 (p = 9.4 x 10^{-19}) (A)	
	DBP (European)	β = 0.25 (p = 1.38 x 10^{-17}) (A)	Surendran P. et al., 2016
	SBP (European)	β = 0.34 (p = 6.06 x 10^{-13}) (A)	
	Hypertension (European)	Z score = 6.07 (p = 1.28 x 10^{-9}) (A)	
	DBP (trans-ethnic data)	β = 0.25 (p = 2.60 x 10^{-19}) (A)	
	SBP (trans-ethnic data)	β = 0.35 (p = 9.94 x 10^{-19}) (A)	
	Hypertension (trans-ethnic data)	Z score = 6.17 (p = 6.82 x 10^{-10}) (A)	
rs2681472 *ATP2B1* (12: 89615182)	DBP	β = 0.50 (p = 1.47×10^{-9}) (A)	Levy D. et al., 2009
	Hypertension	β = 0.15 (p = 1.75×10^{-11}) (A)	
	Coronary artery disease	OR = 1.08 (p = 6.17×10^{-11}) (G)	Nikpay M., et al., 2015

	Myocardial infarction	OR = 1.08 (p = 6.03×10^{-9}) (G)	
	MAP	β = −0.59 (p = 1.1×10^{-21}) (G)	Liu C. et al., 2016
	SBP	β = −0.85 (p = 1.3×10^{-21}) (G)	
	DBP	β = −0.47 (p = 3.7×10^{-17}) (G)	
	Hypertension	β = −0.033 (p = 3.5×10^{-8}) (G)	
	Coronary artery disease	OR = 1.07 (p = 1×10^{-21}) (G)	Nelson C. P., et al., 2017
	SBP	β = 0.72 (p = 1.06×10^{-20}) (A)	Takeuchi F., et al., 2018
	DBP	β = 0.33 (p = 3.77×10^{-11}) (A)	
	MAP	β = 0.46 (p = 5.05×10^{-17}) (A)	
	PP	β = 0.40 (p = 5.49×10^{-14}) (A)	
	Hypertension	β = 0.07(p = 1.41×10^{-6}) (A)	
	Coronary artery disease (trans-ethnic data)	β = 0.060 (p= 6.8×10^{-25}) (G)	Koyama S., et al., 2020
	Coronary artery disease (Japanese)	β = 0.068 (p= 2.6×10^{-11}) (G)	
	Myocardial infarction	OR = 1.07 (p = 1.3×10^{-12}) (G)	Hartiala J. A., et al., 2020
	Myocardial infarction	β = 0.071 (p= 1.17×10^{-11}) (G)	Sakaue S. et al., 2021
rs8068318 *TBX2/ TBX2-AS1* (17:61406405)	MAP	β = −0.28 (p = 2.0×10^{-8}) (C)	Liu C. et al., 2016
	SBP	β = −0.42 (p = 3.9×10^{-17}) (C)	
	Hypertension	Z score = −6.96 (p = 3.0×10^{-12}) (C)	
	DBP	β = −0.26 (p = 3.0×10^{-18}) (C)	
	SBP (European)	β = 0.42 (p= 1.3×10^{-15}) (T)	Surendran P. et al., 2016
	DBP (European)	β = 0.26 (p= 1.95×10^{-16}) (T)	
	Hypertension (European)	Z score = 6.97 (p = 3.21×10^{-12}) (T)	
	DBP (trans-ethnic data)	β = 0.25 (p= 2.75×10^{-18}) (T)	
	SBP (trans-ethnic data)	β = 0.41 (p= 2.3×10^{-17}) (T)	
	Hypertension (trans-ethnic data)	Z score = 6.96 (p = 3.43×10^{-12}) (T)	
rs167479 *RGL3* (19:11416089)	SBP	β = 0.41 (p = 1.6×10^{-21}) (G)	Hoffmann T. J. et al., 2017
	DBP	β = 0.25 (p = 4.3×10^{-22}) (G)	
	PP	β = 0.18 (p = 3.2×10^{-8}) (G)	
	MAP	β = −0.30 (p = 7.3×10^{-11}) (T)	Liu C. et al., 2016
	Hypertension	Z score = −7.72 (p = 1.2×10^{-14}) (T)	
	SBP	β = −0.45 (p = 1.0×10^{-23}) (T)	
	DBP	β = −0.30 (p = 4.2×10^{-28}) (T)	
	DBP (European)	β= −0.33 (p = 1.99×10^{-31}) (T)	Surendran P. et al., 2016
	SBP (European)	β= −0.50 (p =1.49×10^{-26}) (T)	
	Hypertension (European)	Z score = −7.86 (p = 4.01×10^{-15}) (T)	

	DBP (trans-ethnic data)	β= −0.31 (p = 2.76 x 10^{-32}) (T)	
	SBP (trans-ethnic data)	β= −0.47 (p = 8.64 x 10^{-27}) (T)	
	Hypertension (trans-ethnic data)	Z score = −7.88 (p = 3.37 x 10^{-15}) (T)	
	SBP	β = −0.41 (p = 4.32 x 10 $^{-36}$) (T)	Giri A. et el. 2018
	Hypertension	β = 0.05 (p = 8.0 x 10^{-16}) (G)	German C. A., 2019
	Hypertension	OR = 0.84 – 0.92 (p = 2.37 x 10^{-8}) (T)	Jeong H. et al., 2020
	SBP	β = −0.027 (p = 3.24 x 10^{-46}) (T)	Sakaue S. et al., 2021
	DBP	β = −0.026 (p = 2.24 x 10^{-48}) (T)	
	PP	β = −0.016 (p = 7.72 x 10^{-18}) (T)	
	MAP	β = −0.028 (p = 3.64 x 10^{-48}) (T)	

Notes: SBP - systolic blood pressure; DBP - diastolic blood pressure; MAP - mean arterial pressure; PP - pulse blood pressure; z-score - standard scores, β – effect, OR – odds ratio, p – significance level.
