# Supplementary material for: Sex-Specific Features of the Correlation between GWAS-Noticeable Polymorphisms and Hypertension in Europeans of Russia
Source: Int J Mol Sci. 2023 Apr 25;24(9):7799. doi: 10.3390/ijms24097799 (PMC10178435; doi:10.3390/ijms24097799)
Supplement: Supplementary file 1 [file ijms-24-07799-s001.zip › --Suppl table S2.pdf]

Supplementary table S2. The allele and genotype frequencies of the studied SNPs in the HTN and control groups of female.

Gene (SNP, major/minor alleles)	Minor allele frequency,%	Genotype distribution*	H_o	H_e	P_{HWE}
HTN patients (n=375)					
AC026703.1 (rs1173771,G/A)	44.64	67/174/104	0.504	0.494	0.745
HFE (rs1799945,C/G)	17.08	10/105/251	0.287	0.283	1.000
BAG6 (rs805303,G/A)	34.65	48/150/157	0.423	0.453	0.200
PLCE1 (rs932764,A/G)	48.39	86/158/97	0.463	0.500	0.193
OBFC1 (rs4387287,C/A)	22.69	20/107/197	0.330	0.351	0.418
ARHGAP42 (rs633185,C/G)	27.16	33/135/202	0.365	0.396	0.148
CERS5 (rs7302981,G/A)	42.48	64/160/115	0.472	0.489	0.578
ATP2B1 (rs2681472,A/G)	14.96	5/95/251	0.271	0.254	0.296
TBX2 (rs8068318,T/C)	26.44	26/123/182	0.372	0.389	0.399
RGL3 (rs167479,T/G)	51.01	86/183/79	0.526	0.500	0.391
Control group (n=209)					
AC026703.1 (rs1173771,G/A)	46.97	40/106/52	0.535	0.498	0.321
HFE (rs1799945,C/G)	22.46	9/75/123	0.362	0.348	0.691
BAG6 (rs805303,G/A)	34.88	24/95/86	0.463	0.454	0.878
PLCE1 (rs932764,A/G)	49.75	48/106/49	0.522	0.500	0.576
OBFC1 (rs4387287,C/A)	23.24	12/62/111	0.335	0.357	0.411
ARHGAP42 (rs633185,C/G)	26.33	14/81/112	0.391	0.388	1.000
CERS5 (rs7302981,G/A)	38.01	30/89/77	0.454	0.471	0.649
ATP2B1 (rs2681472,A/G)	15.50	5/52/143	0.260	0.262	1.000
TBX2 (rs8068318,T/C)	25.38	13/75/111	0.377	0.379	1.000
RGL3 (rs167479,T/G)	47.52	48/96/58	0.475	0.499	0.485

Note: * minor allele homozygotes / heterozygotes / major allele homozygotes
