# Supplementary material for: Sex-Specific Features of the Correlation between GWAS-Noticeable Polymorphisms and Hypertension in Europeans of Russia
Source: Int J Mol Sci. 2023 Apr 25;24(9):7799. doi: 10.3390/ijms24097799 (PMC10178435; doi:10.3390/ijms24097799)
Supplement: Supplementary file 1 [file ijms-24-07799-s001.zip › --Suppl table S3.pdf]

Supplementary table S3. Genotype combinations associated with HTN in male*

Model	N	Genotype combinations	*beta*	P	Risk, High/Low
Three-order interaction models					
1	1	rs805303 GG *BAG6* × rs1173771 GA *AC026703.1* × rs4387287 CA *OBFC1*	-1.14	0.047	L
	2	rs805303 GG *BAG6* × rs1173771 GA *AC026703.1* × rs4387287 AA *OBFC1*	-0.74	0.023	L
	3	rs805303 GG *BAG6* × rs1173771 AA *AC026703.1* × rs4387287 AA *OBFC1*	2.29	0.029	H
2	4	rs932764 AA *PLCE1* × rs7302981 GG *CERS5* × rs1799945 CC *HFE*	-1.08	0.044	L
	5	rs932764 GG *PLCE1* × rs7302981 AA *CERS5* × rs1799945 CC *HFE*	-0.98	0.008	L
	6	rs932764 GG *PLCE1* × rs7302981 GA *CERS5* × rs1799945 CG *HFE*	-1.08	0.012	L
3	7	rs7302981 GA *CERS5* × rs1173771 GA *AC026703.1* × rs167479 TT *RGL3*	-0.68	0.019	L
	8	rs7302981 AA *CERS5* × rs1173771 AA *AC026703.1* × rs167479 TG *RGL3*	-0.91	0.036	L
	9	rs7302981 GG *CERS5* × rs1173771 GA *AC026703.1* × rs167479 GG *RGL3*	-0.88	0.041	L
Four-order interaction models					
1	1	rs932764 GG *PLCE1* × rs7302981 GG *CERS5* × rs1799945 CC *HFE* × rs8068318 TC *TBX2*	-1.75	0.047	L
	2	rs932764 AG *PLCE1* × rs7302981 AA *CERS5* × rs1799945 CC *HFE* × rs8068318 CC *TBX2*	-0.69	0.032	L
	3	rs932764 GG *PLCE1* × rs7302981 AA *CERS5* × rs1799945 CC *HFE* × rs8068318 CC *TBX2*	-1.69	0.001	L
	4	rs932764 GG *PLCE1* × rs7302981 GA *CERS5* × rs1799945 CG *HFE* × rs8068318 CC *TBX2*	-2.13	0.002	L
2	5	rs932764 AA *PLCE1* × rs7302981 GG *CERS5* × rs1799945 CC *HFE* × rs1173771 GA *AC026703.1*	-2.01	0.019	L
	6	rs932764 GG *PLCE1* × rs7302981 AA *CERS5* × rs1799945 CC *HFE* × rs1173771 GA *AC026703.1*	-1.48	0.004	L
	7	rs932764 GG *PLCE1* × rs7302981 GA *CERS5* × rs1799945 CG *HFE* × rs1173771 AA *AC026703.1*	-1.85	0.005	L
3	8	rs932764 AG *PLCE1* × rs8068318 CC *TBX2* × rs805303 AA *BAG6* × rs1173771 GG *AC026703.1*	-1.63	0.015	L

	9	rs932764 GG *PLCE1* × rs8068318 TC *TBX2* × rs805303 GG *BAG6* × rs1173771 GA *AC026703.1*	-2.63	0.017	L
	10	rs932764 GG *PLCE1* × rs8068318 CC *TBX2* × rs805303 GA *BAG6* × rs1173771 AA *AC026703.1*	-1.55	0.009	L
4	11	rs7302981 AA *CERS5* × rs1799945 CC *HFE* × rs805303 GG *BAG6* × rs167479 TG *RGL3*	-1.38	0.016	L
	12	rs7302981 GG *CERS5* × rs1799945 CC *HFE* × rs805303 GG *BAG6* × rs167479 GG *RGL3*	-2.24	0.042	L
	13	rs7302981 GG *CERS5* × rs1799945 CC *HFE* × rs805303 AA *BAG6* × rs167479 GG *RGL3*	-1.33	0.050	L
	14	rs7302981 GA *CERS5* × rs1799945 CG *HFE* × rs805303 AA *BAG6* × rs167479 GG *RGL3*	-1.62	0.011	L

* Genotype combinations are derived from the interaction models obtained by the MB-MDR method and described in Tables 3
