# Supplementary material for: Sex-Specific Features of the Correlation between GWAS-Noticeable Polymorphisms and Hypertension in Europeans of Russia
Source: Int J Mol Sci. 2023 Apr 25;24(9):7799. doi: 10.3390/ijms24097799 (PMC10178435; doi:10.3390/ijms24097799)
Supplement: Supplementary file 1 [file ijms-24-07799-s001.zip › --Suppl table S4.pdf]

Supplementary table S4. Genotype combinations associated with HTN in female*

Model	N	Genotype combinations	*beta*	P	Risk, High/Low
Two-order interaction models					
1	1	rs8068318 TC *TBX2* × rs167479 TG *RGL3*	-0.50	0.025	L
	2	rs8068318 CC *TBX2* × rs167479 TG *RGL3*	0.49	0.016	H
	3	rs8068318 CC *TBX2* × rs167479 GG *RGL3*	-0.77	0.002	L
2	4	rs932764 AG *PLCE1* × rs1799945 CG *HFE*	-0.62	0.015	L
	5	rs932764 AG *PLCE1* × rs1799945 GG *HFE*	-1.91	0.009	L
Three-order interaction models					
1	1	rs1799945 CC *HFE* × rs8068318 TC *TBX2* × rs167479 TG *RGL3*	-0.52	0.047	L
	2	rs1799945 CC *HFE* × rs8068318 CC *TBX2* × rs167479 TG *RGL3*	0.99	0.0002	H
	3	rs1799945 GG *HFE* × rs8068318 CC *TBX2* × rs167479 TG *RGL3*	-1.59	0.040	L
	4	rs1799945 CC *HFE* × rs8068318 TC *TBX2* × rs167479 GG *RGL3*	1.35	0.021	H
	5	rs1799945 CG *HFE* × rs8068318 CC *TBX2* × rs167479 GG *RGL3*	-1.21	0.005	L
2	6	rs2681472 AA *ATP2B1* × rs8068318 TC *TBX2* × rs167479 TG *RGL3*	-0.76	0.003	L
	7	rs2681472 AA *ATP2B1* × rs8068318 CC *TBX2* × rs167479 TG *RGL3*	0.69	0.003	H
	8	rs2681472 AA *ATP2B1* × rs8068318 CC *TBX2* × rs167479 GG *RGL3*	-0.69	0.010	L
3	9	rs932764 GG *PLCE1* × rs7302981 GA *CERS5* × rs805303 GA *BAG6*	-1.25	0.003	L
	10	rs932764 AA *PLCE1* × rs7302981 GA *CERS5* × rs805303 AA *BAG6*	0.89	0.038	H
	11	rs932764 AG *PLCE1* × rs7302981 AA *CERS5* × rs805303 AA *BAG6*	-1.21	0.0008	L
4	12	rs932764 AA *PLCE1* × rs805303 AA *BAG6* × rs4387287 AA *OBFC1*	1.30	0.004	H
5	13	rs932764 GG *PLCE1* × rs805303 AA *BAG6* × rs4387287 CA *OBFC1*	0.51	0.026	H

	14	rs932764 GG *PLCE1* × rs805303 GA *BAG6* × rs4387287 AA *OBFC1*	-0.97	0.031	L
	15	rs932764 GG *PLCE1* × rs805303 AA *BAG6* × rs4387287 AA *OBFC1*	-0.58	0.040	L
6	16	rs2681472 AG *ATP2B1* × rs805303 GG *BAG6* × rs1173771 GA *AC026703.1*	-1.77	0.039	L
	17	rs2681472 AG *ATP2B1* × rs805303 GA *BAG6* × rs1173771 GA *AC026703.1*	-1.35	0.004	L
	18	rs2681472 AG *ATP2B1* × rs805303 AA *BAG6* × rs1173771 GA *AC026703.1*	1.40	0.005	H
7	19	rs932764 AG *PLCE1* × rs8068318 TC *TBX2* × rs167479 TG *RGL3*	-0.85	0.003	L
	20	rs932764 AA *PLCE1* × rs8068318 CC *TBX2* × rs167479 TG *RGL3*	0.97	0.027	H
	21	rs932764 AA *PLCE1* × rs8068318 TC *TBX2* × rs167479 GG *RGL3*	2.45	0.024	H
	22	rs932764 AG *PLCE1* × rs8068318 CC *TBX2* × rs167479 GG *RGL3*	-0.86	0.006	L
8	23	rs932764 AG *PLCE1* × rs1799945 CG *HFE* × rs805303 AA *BAG6*	-1.27	0.0009	L
	24	rs932764 AG *PLCE1* × rs1799945 GG *HFE* × rs805303 AA *BAG6*	-1.97	0.032	L
9	25	rs932764 AG *PLCE1* × rs1799945 CC *HFE* × rs8068318 CC *TBX2*	0.67	0.004	H
	26	rs932764 AG *PLCE1* × rs1799945 CG *HFE* × rs8068318 CC *TBX2*	-0.82	0.010	L
	27	rs932764 AG *PLCE1* × rs1799945 CG *HFE* × rs8068318 CC *TBX2*	-2.87	0.012	L
10	28	rs932764 AG *PLCE1* × rs1799945 GG *HFE* × rs167479 TG *RGL3*	-2.18	0.012	L
	29	rs932764 AG *PLCE1* × rs1799945 CG *HFE* × rs167479 GG *RGL3*	-1.09	0.010	L
Four-order interaction models					
1	1	rs932764 AG *PLCE1* × rs8068318 TC *TBX2* × rs1173771 GA *AC026703.1* × rs167479 TG *RGL3*	-1.10	0.008	L
	2	rs932764 AA *PLCE1* × rs8068318 CC *TBX2* × rs1173771 GG *AC026703.1* × rs167479 GG *RGL3*	-2.10	0.050	L
	3	rs932764 AG *PLCE1* × rs8068318 CC *TBX2* × rs1173771 GA *AC026703.1* × rs167479 GG *RGL3*	-0.77	0.050	L
2	4	rs1799945 CC *HFE* × rs8068318 CC *TBX2* × rs805303 GG *BAG6* × rs167479 TG *RGL3*	1.57	0.044	H
	5	rs1799945 CC *HFE* × rs8068318 TC *TBX2* × rs805303 GA *BAG6* × rs167479 TG *RGL3*	-0.72	0.033	L
	6	rs1799945 CC *HFE* × rs8068318 CC *TBX2* × rs805303 AA *BAG6* × rs167479 TG *RGL3*	0.82	0.026	H
	7	rs1799945 GG *HFE* × rs8068318 CC *TBX2* × rs805303 AA *BAG6* × rs167479 TG *RGL3*	-2.53	0.038	L

	8	rs1799945 CG *HFE* × rs8068318 TC *TBX2* × rs805303 GA *BAG6* × rs167479 GG *RGL3*	-1.57	0.013	L
	9	rs1799945 CG *HFE* × rs8068318 CC *TBX2* × rs805303 AA *BAG6* × rs167479 GG *RGL3*	-1.82	0.046	L
3	10	rs932764 GG *PLCE1* × rs1799945 CC *HFE* × rs1173771 GA *AC026703.1* × rs167479 TT *RGL3*	-1.64	0.025	L
	11	rs932764 AG *PLCE1* × rs1799945 GG *HFE* × rs1173771 GA *AC026703.1* × rs167479 TG *RGL3*	-2.79	0.016	L
	12	rs932764 GG *PLCE1* × rs1799945 CG *HFE* × rs1173771 AA *AC026703.1* × rs167479 TG *RGL3*	-1.98	0.015	L
	13	rs932764 AA *PLCE1* × rs1799945 GG *HFE* × rs1173771 GG *AC026703.1* × rs167479 GG *RGL3*	-2.29	0.033	L
4	14	rs2681472 AA *ATP2B1* × rs1799945 CC *HFE* × rs8068318 TC *TBX2* × rs167479 TG *RGL3*	-0.84	0.005	L
	15	rs2681472 AA *ATP2B1* × rs1799945 CC *HFE* × rs8068318 CC *TBX2* × rs167479 TG *RGL3*	0.97	0.0007	H
	16	rs2681472 AG *ATP2B1* × rs1799945 CG *HFE* × rs8068318 CC *TBX2* × rs167479 TG *RGL3*	-1.05	0.031	L
	17	rs2681472 AA *ATP2B1* × rs1799945 GG *HFE* × rs8068318 CC *TBX2* × rs167479 TG *RGL3*	-1.83	0.048	L
	18	rs2681472 AA *ATP2B1* × rs1799945 CC *HFE* × rs8068318 TC *TBX2* × rs167479 GG *RGL3*	1.36	0.042	H
	19	rs2681472 AA *ATP2B1* × rs1799945 CG *HFE* × rs8068318 CC *TBX2* × rs167479 GG *RGL3*	-1.23	0.017	L
5	20	rs1799945 CC *HFE* × rs8068318 CC *TBX2* × rs1173771 GG *AC026703.1* × rs167479 TG *RGL3*	1.57	0.039	H
	21	rs1799945 CC *HFE* × rs8068318 CC *TBX2* × rs1173771 GA *AC026703.1* × rs167479 TG *RGL3*	0.69	0.024	H
	22	rs1799945 GG *HFE* × rs8068318 CC *TBX2* × rs1173771 GA *AC026703.1* × rs167479 TG *RGL3*	-2.57	0.032	L
	23	rs1799945 CG *HFE* × rs8068318 CC *TBX2* × rs1173771 AA *AC026703.1* × rs167479 TG *RGL3*	-1.04	0.049	L
	24	rs1799945 CC *HFE* × rs8068318 CC *TBX2* × rs1173771 GG *AC026703.1* × rs167479 GG *RGL3*	-1.33	0.011	L
	25	rs1799945 CG *HFE* × rs8068318 CC *TBX2* × rs1173771 GA *AC026703.1* × rs167479 GG *RGL3*	-1.19	0.026	L
6	26	rs932764 AG *PLCE1* × rs1799945 CC *HFE* × rs8068318 CC *TBX2* × rs167479 TT *RGL3*	1.24	0.027	H
	27	rs932764 AG *PLCE1* × rs1799945 CC *HFE* × rs8068318 TC *TBX2* × rs167479 TG *RGL3*	-0.83	0.012	L
	28	rs932764 AG *PLCE1* × rs1799945 CC *HFE* × rs8068318 CC *TBX2* × rs167479 TG *RGL3*	1.02	0.004	H
	29	rs932764 GG *PLCE1* × rs1799945 CG *HFE* × rs8068318 CC *TBX2* × rs167479 TG *RGL3*	-0.98	0.047	L
	30	rs932764 AG *PLCE1* × rs1799945 GG *HFE* × rs8068318 CC *TBX2* × rs167479 TG *RGL3*	-2.88	0.012	L
	31	rs932764 AG *PLCE1* × rs1799945 CG *HFE* × rs8068318 CC *TBX2* × rs167479 GG *RGL3*	-1.70	0.003	L

7	32	rs1799945 CC *HFE* × rs8068318 CC *TBX2* × rs4387287 AA *OBFC1* × rs167479 TT *RGL3*	1.97	0.009	H
	33	rs1799945 CC *HFE* × rs8068318 CC *TBX2* × rs4387287 AA *OBFC1* × rs167479 TG *RGL3*	1.11	0.0004	H
	34	rs1799945 CG *HFE* × rs8068318 CC *TBX2* × rs4387287 AA *OBFC1* × rs167479 GG *RGL3*	-1.07	0.024	L
8	35	rs2681472 AG *ATP2B1* × rs633185 CG *ARHGAP42* × rs7302981 GA *CERS5* × rs805303 GA *BAG6*	-1.66	0.008	L
	36	rs2681472 AG *ATP2B1* × rs633185 GG *ARHGAP42* × rs7302981 GA *CERS5* × rs805303 GA *BAG6*	-1.31	0.012	L
	37	rs2681472 AA *ATP2B1* × rs633185 GG *ARHGAP42* × rs7302981 AA *CERS5* × rs805303 AA *BAG6*	-0.87	0.027	L
9	38	rs2681472 AA *ATP2B1* × rs633185 GG *ARHGAP42* × rs8068318 TC *TBX2* × rs167479 TG *RGL3*	-0.99	0.003	L
	39	rs2681472 AA *ATP2B1* × rs633185 GG *ARHGAP42* × rs8068318 CC *TBX2* × rs167479 TG *RGL3*	0.65	0.017	H
	40	rs2681472 AA *ATP2B1* × rs633185 CG *ARHGAP42* × rs8068318 TC *TBX2* × rs167479 GG *RGL3*	1.61	0.049	H
	41	rs2681472 AA *ATP2B1* × rs633185 CG *ARHGAP42* × rs8068318 CC *TBX2* × rs167479 GG *RGL3*	-1.08	0.006	L
10	42	rs2681472 AG *ATP2B1* × rs8068318 CC *TBX2* × rs1173771 GA *AC026703.1* × rs167479 TT *RGL3*	-1.34	0.036	L
	43	rs2681472 AA *ATP2B1* × rs8068318 TC *TBX2* × rs1173771 GA *AC026703.1* × rs167479 TG *RGL3*	-0.94	0.004	L
	44	rs2681472 AA *ATP2B1* × rs8068318 CC *TBX2* × rs1173771 AA *AC026703.1* × rs167479 TG *RGL3*	1.16	0.042	H
	45	rs2681472 AG *ATP2B1* × rs8068318 CC *TBX2* × rs1173771 AA *AC026703.1* × rs167479 TG *RGL3*	-1.03	0.043	L
	46	rs2681472 AA *ATP2B1* × rs8068318 CC *TBX2* × rs1173771 GG *AC026703.1* × rs167479 GG *RGL3*	-1.17	0.024	L
11	47	rs633185 GG *ARHGAP42* × rs932764 GG *PLCE1* × rs7302981 GA *CERS5* × rs805303 GA *BAG6*	-3.11	0.003	L
	48	rs633185 GG *ARHGAP42* × rs932764 AA *PLCE1* × rs7302981 GA *CERS5* × rs805303 AA *BAG6*	1.32	0.037	H
	49	rs633185 GG *ARHGAP42* × rs932764 AG *PLCE1* × rs7302981 AA *CERS5* × rs805303 AA *BAG6*	-2.00	0.0002	L
12	50	rs932764 AA *PLCE1* × rs7302981 GA *CERS5* × rs1799945 CC *HFE* × rs167479 TT *RGL3*	-1.70	0.050	L
	51	rs932764 AA *PLCE1* × rs7302981 GA *CERS5* × rs1799945 CG *HFE* × rs167479 TG *RGL3*	-1.58	0.004	L
	52	rs932764 GA *PLCE1* × rs7302981 GA *CERS5* × rs1799945 CG *HFE* × rs167479 GG *RGL3*	-1.07	0.046	L
13	53	rs7302981 AA *CERS5* × rs1799945 CC *HFE* × rs8068318 TC *TBX2* × rs167479 TG *RGL3*	-0.98	0.022	L
	54	rs7302981 GA *CERS5* × rs1799945 CC *HFE* × rs8068318 CC *TBX2* × rs167479 TG *RGL3*	0.69	0.035	H
	55	rs7302981 AA *CERS5* × rs1799945 CC *HFE* × rs8068318 CC *TBX2* × rs167479 TG *RGL3*	1.13	0.025	H

	56	rs7302981 AA *CERS5* × rs1799945 CC *HFE* × rs8068318 CC *TBX2* × rs167479 GG *RGL3*	-1.48	0.009	L
	57	rs7302981 GA *CERS5* × rs1799945 CG *HFE* × rs8068318 CC *TBX2* × rs167479 GG *RGL3*	-1.49	0.024	L
14	58	rs633185 GG *ARHGAP42* × rs7302981 AA *CERS5* × rs8068318 TC *TBX2* × rs167479 TG *RGL3*	-1.17	0.007	L
	59	rs633185 CG *ARHGAP42* × rs7302981 GA *CERS5* × rs8068318 CC *TBX2* × rs167479 GG *RGL3*	-0.99	0.026	L
	60	rs633185 GG *ARHGAP42* × rs7302981 AA *CERS5* × rs8068318 CC *TBX2* × rs167479 GG *RGL3*	-1.50	0.011	L

* Genotype combinations are derived from the interaction models obtained by the MB-MDR method and described in Tables 3
